# Impact of polymorphism on the optoelectronic properties of a low-bandgap semiconducting polymer

**DOI:** 10.1038/s41467-019-10519-z

**Published:** 2019-06-28

**Authors:** Mengmeng Li, Ahmed Hesham Balawi, Pieter J. Leenaers, Lu Ning, Gaël H. L. Heintges, Tomasz Marszalek, Wojciech Pisula, Martijn M. Wienk, Stefan C. J. Meskers, Yuanping Yi, Frédéric Laquai, René A. J. Janssen

**Affiliations:** 1 Molecular Materials and Nanosystems, Institute for Complex Molecular Systems, Eindhoven University of Technology, P.O. Box 513, 5600 MB Eindhoven, The Netherlands; 2 Dutch Institute for Fundamental Energy Research, De Zaale 20, 5612 AJ Eindhoven, The Netherlands; 3 KAUST Solar Center (KSC), Physical Sciences and Engineering Division (PSE, Material Science and Engineering Program (MSE)), King Abdullah University of Science and Technology (KAUST), Thuwal, 23955-6900 Saudi Arabia; 4 Beijing National Laboratory for Molecular Science, Key Laboratory of Organic Solids, Institute of Chemistry, Chinese Academy of Sciences, 100190 Beijing, People’s Republic of China; 5 Institute for Materials Research (IMO-IMOMEC), Design and Synthesis of Organic Semiconductors (DSOS), Hasselt University, Agoralaan, 3590 Diepenbeek, Belgium; 6 Max Planck Institute for Polymer Research, Ackermannweg 10, 55128 Mainz, Germany; 7 Faculty of Chemistry, Department of Molecular Physics, Lodz University of Technology, Zeromskiego, 116, 90-924 Lodz, Poland

**Keywords:** Conjugated polymers, Electronic devices

## Abstract

Polymorphism of organic semiconducting materials exerts critical effects on their physical properties such as optical absorption, emission and electrical conductivity, and provides an excellent platform for investigating structure–property relations. It is, however, challenging to efficiently tune the polymorphism of conjugated polymers in aggregated, semi-crystalline phases due to their conformational freedom and anisotropic nature. Here, two distinctly different semi-crystalline polymorphs (*β*_1_ and *β*_2_) of a low-bandgap diketopyrrolopyrrole polymer are formed through controlling the solvent quality, as evidenced by spectroscopic, structural, thermal and charge transport studies. Compared to *β*_1_, the *β*_2_ polymorph exhibits a lower optical band gap, an enhanced photoluminescence, a reduced π-stacking distance, a higher hole mobility in field-effect transistors and improved photocurrent generation in polymer solar cells. The *β*_1_ and *β*_2_ polymorphs provide insights into the control of polymer self-organization for plastic electronics and hold potential for developing programmable ink formulations for next-generation electronic devices.

## Abstract (web summary)

Tuning polymorphism of conjugated polymers, though a promising method for studying and controlling the structure-property relations in these materials remains a challenge. Here, the authors identify two aggregated semi-crystalline polymorphs of a low-bandgap diketopyrrolopyrrole-based polymer.

## Introduction

Polymorphism is the ability of a compound to adopt multiple packing motifs in the solid or aggregated state and has important consequences in chemistry, physics, material science, and biology^1^. Among organic semiconducting materials, considerable progress in understanding polymorphism has been made using single crystals of conjugated small molecules^2–6^. It was found that a slight change in crystal packing critically affects the charge transport in electronic devices^6^. Traditionally the structure-property relations of organic semiconductors are mainly studied via chemical modifications such as regioregularity control and side chain engineering^7,8^. In comparison, polymorphs provide an ideal platform for such investigations by excluding the influence of chemical modification. Polymorphism of conjugated semiconducting polymers has been reported for few polymers^9–17^.

In most conjugated polymers, an amorphous “spaghetti-like” phase (*α*) typically coexists with an aggregated phase (*β*) composed of relatively ordered crystalline regions^18^. The *β* phase originates from strong intermolecular interactions between functional units or aromatic rings in the polymer backbone^19^. The transition between the amorphous *α* and semi-crystalline *β* phases of conjugated polymers, such as in polyfluorenes, can be realized via thermal treatment^20^ or by using mixtures of good/poor solvents^21–23^. A comprehensive investigation of polymorphism of the semi-crystalline *β* phases of conjugated polymers has not yet been achieved. One prominent example that grants access to two different semi-crystalline polymorphs is formed by poly(3-alkylthiophene)s, whose solid state structure can be controlled by the evaporation rate of solvent^24^, exposure to solvent vapors^25^, molecular weight^26^, and thermal annealing^27^. The first polymorph found in most occasions exhibits an end-to-end packing of the lamellae, while the second one has interdigitated side chains with a reduced lamellar spacing, as confirmed using X-ray measurements^28^. From the aspect of spectroscopy, two H-type aggregates in solution were identified at extremely low temperature for poly(3-hexylthiophene) by Franck-Condon analyses of emission spectra^29^. Another example of polymorphism has been found for the naphthalene diimide based copolymer (P(NDI2OD-T2)) that shows an extra absorption peak at longer wavelengths when dissolved in a poor solvent, indicating the formation of a secondary aggregated phase^19^. Other signs of polymorphism were found for conjugated polymers, blended with polyethylene oxide (PEO) which afforded differences in the optical absorption before and after treatment with water.^30^ Generally, the magnitude of the changes in optoelectronic or thermal properties depends on the structural differences between the polymorphs. Polymorphism in aggregated phases can also be obtained in solid films, but post-treatment or particular growth methods are prerequisites^9,19^. However, these reports lack detailed spectroscopic and structural characterizations, which could reveal the role of polymorphism in the structure-property relations and, more importantly, in the optoelectronic property in electronic devices.

In this contribution, two aggregated, semi-crystalline phases (*β*_1_ and *β*_2_) of a diketopyrrolopyrrole (DPP) based polymer^31^ are generated by precisely tuning the solvent quality. Both polymorphs can be formed in solution and in the solid state. The *β*_2_ phase possess a lower optical band gap, demonstrating a more delocalized wave function. The steady-state and time-resolved photoluminescence (PL) of polymer solutions reveal that the three phases, namely *α*, *β*_1_ and *β*_2_, can coexist with excited state lifetimes of 29 ps, 78 ps, and 129 ps, respectively. The extended chain conformation of the newly induced *β*_2_ phase in solution is retained in thin films, as confirmed by both UV-vis-NIR absorption and PL measurements. Compared to *β*_1_, the *β*_2_ phase possesses a slightly smaller π-stacking distance but has identical lamellar spacing. The field-effect mobility of the *β*_2_ phase is higher than that of *β*_1_, likely due to the reduced π–π stacking. In bulk heterojunction solar cells, a clear contribution of the *β*_2_ polymorph to the external quantum efficiency (EQE) is observed via a long-wavelength peak. This study proposes a simple approach for tuning the polymorphism of conjugated polymers in both solution and solid state, and the characterization provides insights into the structure-property relation of next-generation devices for plastic electronics.

## Results

### Polymorphism in solution

**Fig. 1:**
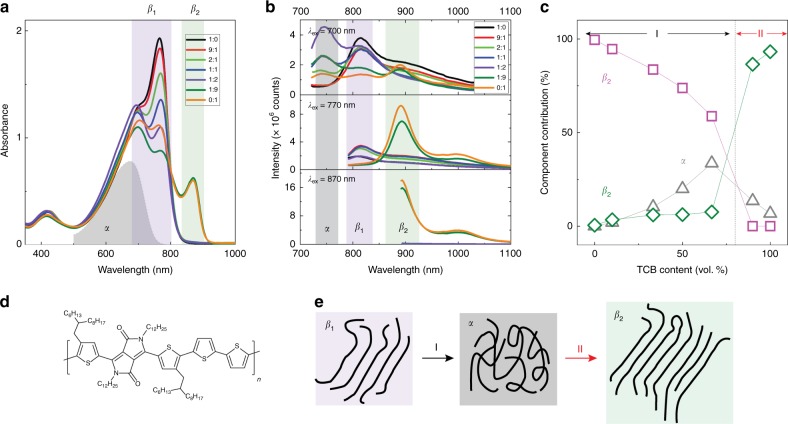
Optical absorption and steady-state photoluminescence spectra in mixed solvents. **a**, **b** UV-vis-NIR absorption and steady-state photoluminescence spectra of D-PDPP4T-HD dissolved in chloroform:1,2,4-trichlorobenzene (CF:TCB) mixtures. The volume ratio (v/v) of CF and TCB is 1:0, 9:1, 2:1, 1:1, 1:2, 1:9, and 0:1, respectively. The polymer concentration is 0.4 μM in all cases. The gray area in **a** represents the absorption spectrum of the amorphous phase (*α*) in pure TCB at 100 °C (Supplementary Fig. 2). The characteristic peaks of the first (*β*_1_) and the second (*β*_2_) aggregated phases are indicated by purple and green frames, respectively. **c** The component contribution of each phase as a function of the TCB content, as inferred from the multivariate curve resolution alternating least squares deconvolution of the UV-vis-NIR spectra. Process I in **c** indicates the *β*_1_ → *α* transformation when TCB content increases from 0 to 67%; process II indicates the *α* *→* *β*_2_ transformation when the TCB content increases further from 67 to 100%. **d** Chemical structure of D-PDPP4T-HD. **e** Schematic illustration of polymorphism transitions of D-PDPP4T-HD

DPP based polymers are a versatile class of organic semiconducting materials, whose optical properties can be manipulated from small (1.13 eV) to wide band gaps (1.77 eV) by modifying the aromatic substituents and π−conjugated segments^32–34^. Typically, the UV-vis-NIR absorption spectra of DPP based polymers exhibit an intense first (0–0) vibronic absorption band and a less intense vibronic progression at shorter wavelengths (Supplementary Fig. 1), indicative of J-type aggregates^35^. As shown in Fig. 1a, the absorption spectrum of D-PDPP4T-HD shows 0–0 and 0–1 vibronic peaks in dilute chloroform (CF) solution at 770 and 700 nm, respectively, together with a band at 424 nm due to a higher energy electronic state. Compared to the amorphous phase (denoted as *α*) in which the polymer is molecularly dissolved, this aggregated phase is termed *β*_1_. The intensity of the 0–0 peak gradually decreases with the addition of 1,2,4-trichlorobenzene (TCB) to the CF solution, without affecting the 0–1 peak. With increasing TCB content, the steady-state photoluminescence (PL) spectra reveal a reduced emission of the *β*_1_ phase at 816 nm, with a simultaneous increase of the emission of the *α* phase at 744 nm. From the PL spectra we determine that in pure CF the *α* phase concentration is negligible because only *β*_1_ emission is observed with excitation at 700 nm, and emission of *α* phase at 740–750 nm is absent (black line in upper panel of Fig. 1b). These changes suggest a transition from the ordered *β*_1_ phase in CF to the amorphous *α* phase upon adding TCB. When the CF:TCB ratio decreases to 1:1, the intensity of the 0–0 absorption peak becomes identical to that of the 0–1 peak. Surprisingly, when the CF:TCB ratio reaches 1:9, a completely new absorption peak emerges at 870 nm, accompanied by an additional emission peak at 890 nm (Fig. 1a, b). Such change in both absorption and PL points to the formation of a second aggregated phase (*β*_2_). The red-shifted absorption and emission of the *β*_2_ phase suggest that its wave functions are more delocalized, either by a decrease of intrachain disorder or by enhanced interchain interactions. Upon increasing the temperature of solutions containing mainly the *β*_1_ phase or *β*_2_ phase, their UV-vis-NIR absorption spectra exhibit an initial blue shift of the onset of absorption (Supplementary Fig.  2), followed by transformation into a featureless absorption band in a narrow temperature range, characteristic of the *α* phase in which intermolecular interactions are absent, followed at still higher temperature by a gradual spectral shift to lower wavelengths that can be interpreted as being the result of increased conformational freedom (interring rotation) of the polymer backbone.^23,36,37^

To quantify the phase evolution, the UV-vis-NIR absorption spectra recorded for D-PDPP4T-HD solutions as function of CF:TCB ratio are deconvoluted using multivariate curve resolution alternating least squares (MCR-ALS) analysis, as shown in Fig. 1c and Supplementary Fig. 3. By gradually adding TCB to CF from 0 vol.% (1:0) to 66.7 vol.% (1:2), the concentration of the *β*_1_ phase continuously decreases, accompanied by a simultaneous increase of the *α* phase contribution, demonstrating the *β*_1_ → *α* transformation (process I in Fig. 1e). The enhanced emission of *α* (Fig. 1b) confirms this *β*_1_ → *α* transition. Eventually, more TCB (1:9 and 0:1) reduces the contents of the *α* and *β*_1_ phases to the point that they almost cease to be present, while the new *β*_2_ phase appears. At the highest TCB concentrations (1:9 and 0:1), the *β*_2_ phase dominates and only emission of the *β*_2_ phase is observed in the PL spectra (Fig. 1b).

**Fig. 2:**
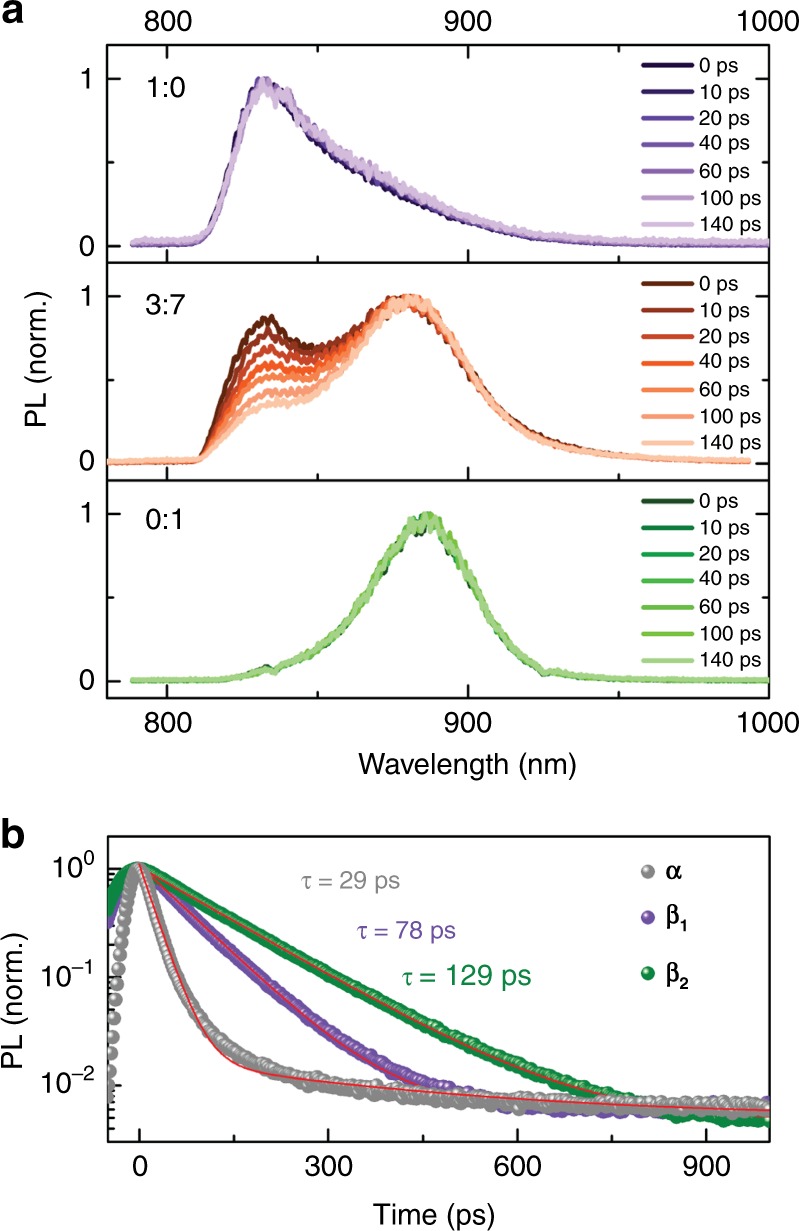
Time-resolved photoluminescence in mixed solvents. **a** Normalized time-resolved photoluminescence (TRPL) spectra recorded at various time delays for a solution of D-PDPP4T-HD in 1:0, 3:7 and 0:1 (v/v) of chloroform:1,2,4-trichlorobenzene (CF:TCB). The polymer concentration is 0.4 µM. Excitation was performed at 800 nm with 80 MHz laser pulse repetition rate. A 808 nm long-pass filter was used to suppress the scattered laser pulse. **b** Emission dynamics of *α* phase from 0:1 solution at 100 °C with *λ*_ex = _400 nm and *λ*_em = _815–865 nm, *β*_1_ phase from 1:0 solution with *λ*_ex = _800 nm and *λ*_em = _825–840 nm, and *β*_2_ phase from 0:1 solution with *λ*_ex = _800 nm and *λ*_em = _870–890 nm

To study the luminescence properties of the three polymorphs of D-PDPP4T-HD, we performed time-resolved photoluminescence (TRPL) measurements on dilute CF:TCB solutions. First, the two aggregated phases, *β*_1_ and *β*_2_, are investigated using excitation at 800 nm, i.e., without exciting the *α* phase. Fig. 2a confirms that in the pure CF and TCB only one phase is present, i.e., only *β*_1_ (*λ*_em_ = 825–840 nm) for 1:0 and only *β*_2_ (*λ*_em_ = 870–890 nm) for 0:1. The corresponding PL kinetics are fitted with mono-exponential decays, and fluorescence lifetimes of 78 ps for *β*_1_ and 129 ps for *β*_2_ (Fig. 2b) are determined. The PL lifetime of *α* phase (29 ps) was determined in a TCB solution at 100 °C (Supplementary Fig. 4).

The possibility of interaction between the two aggregated phases, *β*_1_ and *β*_2_, was investigated by time-resolved PL experiments at the onset of *β*_2_ formation. At a ratio of 3:7 (70% TCB) only a tiny amount of *β*_2_ is seen in the UV-vis-NIR absorption, and *β*_1_ is the dominant aggregated phase (Supplementary Fig. 5). At this critical ratio of 3:7, both emission peaks of *β*_1_ and *β*_2_ are clearly observed in the PL spectrum (Fig. 2a). In spite of the minor population of *β*_2_ at the 3:7 ratio, the PL intensities of *β*_1_ and *β*_2_ are almost the same in the first picoseconds after excitation. This is a consequence of the fact that the PL quantum yield of *β*_2_ is more than 30-fold higher than that of *β*_1_ (Supplementary Fig. 6 and Supplementary Note 1). At the 3:7 ratio, average PL lifetimes of 68 ps (*λ*_em_ = 835 nm) for *β*_1_ and 125 ps (*λ*_em_ = 880 nm) for *β*_2_ are found using bi-exponential fits (Supplementary Fig. 7 and Supplementary Table 1). These values are almost identical to the PL lifetimes of the pure aggregated phases, indicating that there is no, or very little, excited state energy transfer from *β*_1_ to *β*_2_. This indicates that the transformation of *β*_1_ into *β*_2_ does not occur within the aggregated phase (i.e., $\beta_{1} \rightarrow\!\!\!\!\!\!\!\times\; \beta_{2}$), because in such case the close contact between the two phases should result in excited state energy transfer. Instead, the *β*_2_ phase is formed via the molecularly dissolved amorphous *α* phase (i.e., *β*_1_ → *α* → *β*_2_). This is also supported by the temperature dependent UV-vis-NIR absorption spectra that show *β*_1_ → *α* and *β*_2_ → *α* transitions, but no indication of a *β*_2_ → *β*_1_ transformation (Supplementary Fig. 2). Further experiments for different solvent ratios (Supplementary Figs. 7, 8, and 9 and Supplementary Tables 2 and 3) also did not reveal any sign of energy transfer among *α*_,_ *β*_1_ and *β*_2_. This indicates that in solution the phases are beyond the Förster radius (on the order of 5 nm) of each other.

**Fig. 3:**
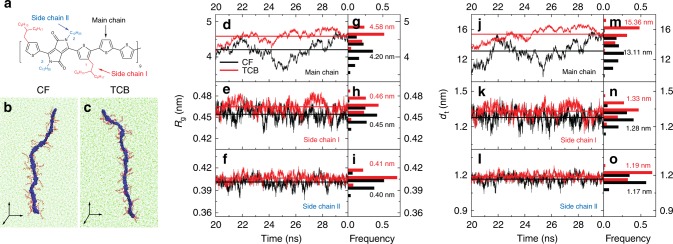
Molecular dynamics simulations. **a** Chemical structure for a D-PDPP4T-HD nonamer. **b**, **c** Snapshots from trajectories of D-PDPP4T-HD nonamer chains in chloroform (CF) and 1,2,4-trichlorobenzene (TCB). **d**, **e**, **f**, Gyration radius (*R*_g_) of main chain, side chain I and side chain II in CF (black) and TCB (red). The straight lines indicate the average values of *R*_g_. **g**, **h**, **i**, Corresponding distributions of **d**, **e**, **f**. **j**, **k**, **l**, Head-to-tail distance (*d*_t_) of main chain, side chain I and side chain II in CF (black) and TCB (red). The straight lines indicate the average values of *d*_t_. **m**, **n**, **o**, Corresponding distributions of **j**, **k**, **l**. The last 10 ns simulation time after equilibration was selected for sampling. If all chains were straight, the *d*_t_ of main chain, side chain I and side chain II would be 18.52, 1.62, and 1.41 nm, respectively

Having established the existence of *β*_1_ and *β*_2_, we sought to reveal the origin of the two aggregated phases. To this aim we employ molecular dynamics simulations for a D-PDPP4T-HD nonamer in CF and TCB (Fig. 3a), following the protocol described in ref. ^38^. The simulation window between 20 and 30 ns is selected for data sampling, because at 20 ns thermal equilibrium has been achieved (Supplementary Fig. 10). In particular, we analyze the conformation of the conjugated chain (main chain), the branched side chain on the thiophene unit (side chain I) and the linear side chain on the DPP unit (side chain II) (Fig. 3b, c). The size of the nonamer is quantitatively evaluated by its gyration radius (*R*_g_) and by the distances between the heads and the tails (*d*_t_) of the main and side chains, as shown in Fig. 3d-o. Compared to CF, the average *R*_g_ of the main chain in TCB is noticeably enhanced from 4.20 nm to 4.58 nm. Moreover, the average value of the main chain *d*_t_ is also increased from 13.11 nm in CF to 15.36 nm in TCB. The increase in both *R*_g_ and *d*_t_ indicates a more extended conformation of main chain caused by TCB. Likewise, both side chains (I and II) follow the same trend, with slightly higher values of *R*_g_ and *d*_t_ in TCB, suggesting that alkyl chains are slightly stretched away from the main chain in the presence of TCB. These results indicate that TCB results in a more extended confirmation of D-PDPP4T-HD than CF. Such extended conformation facilitates polymer crystallization via π–π interaction. This is in excellent agreement with UV-vis-NIR absorption spectra shown in Fig. 1, where the 0–0 peak of *β*_2_ phase is at longer wavelengths, which is generally associated with a more extended excited state wave function via combination of enhanced π–π stacking and increased co-planarity of the heteroaromatic units in the main chain.

### Polymorphism in the solid state

**Fig. 4:**
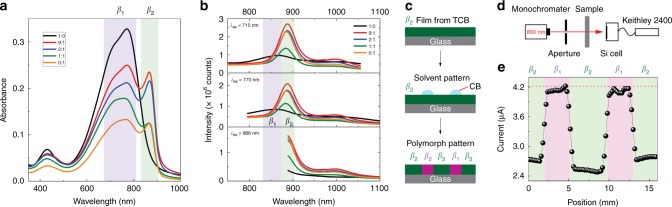
Optical absorption and steady-state emission spectra of thin films. **a** UV-vis-NIR absorption of D-PDPP4T-HD thin films spin-coated from chloroform:1,2,4-trichlorobenzene (CF:TCB) solutions with the polymer concentration of 3 mg mL^−1^. The characteristic peaks for the *β*_1_ and *β*_2_ polymorphs are indicated by purple and green frames, respectively. **b** Corresponding steady-state PL spectra. **c** A polymorph pattern is realized by locally exposing a TCB spin-coated (*β*_2_ phase) film to chlorobenzene (CB). **d** Schematic illustration of the set up used to measure the polymorph pattern, where 860 nm light with a beam radius of 0.5 mm is used. **e** Transmitted light at 860 nm as a function of position. The red dash line (4.22 μA) indicates the current measured for bare glass without polymer film

Interestingly, polymer chains of D-PDPP4T-HD partly resemble their solution polymorph in the solid state. Fig. 4 shows the optical absorption and steady-state PL spectra of D-PDPP4T-HD thin films spin-coated from solutions with various CF:TCB ratios. Small amounts of TCB are able of generating the *β*_2_ phase in the solid state. Only pure CF leads to the formation of a pure *β*_1_ phase. This can be attributed to the much higher boiling point of TCB (214 °C) compared to CF (61 °C) and the corresponding difference in vapor pressure (197 vs. 0.46 mm Hg at 25 °C)^39^. Hence, at the final stage of film formation during spin-coating, a highly concentrated liquid-like film is formed with TCB as the main solvent, since CF has evaporated. At that stage TCB controls the film formation and thus the morphology. Although the emission peak of the *β*_1_ phase is red-shifted from 816 nm for solutions to around 860 nm for solid thin films, the emission peak of *β*_2_ phase remains at the same position (890 nm), as shown in Fig. 4b. Interestingly, the film with the *β*_2_ polymorph can be transformed back into *β*_1_ by processing CF over the film (Supplementary Fig. 11). As shown in Fig. 4c-e this feature enables patterning of the *β*_1_ and *β*_2_ phase in films, creating a location-dependent functionality.

It has been reported that long-term solution aging has a pronounced impact on polymer self-assembly in both solution and solid state^40^. Hence, pure TCB solutions of D-PDPP4T-HD were aged from 2 to 8 days. The solution aging results in a significant increase in the intensity of 0–0 absorption peak of the *β*_2_ phase (Supplementary Fig. 12). The kinetics of aggregate formation from the amorphous *α* phase demonstrate that the growth rate of the *β*_2_ phase in CF:TCB is three orders of magnitude lower than that of the *β*_1_ phase in CF (Supplementary Fig. 13 and Supplementary Table 4). We find that aged solutions affect the solid state in a different way. By aging pure CF solutions for 2 days, the deposited D-PDPP4T-HD thin film shows a clear shoulder at 870 nm, indicating formation of some *β*_2_ phase (Supplementary Fig. 14). On the other hand, aging a CF solution containing TCB has no effect on the thin film UV-vis-NIR spectrum (Supplementary Fig. 15). The difference in aging behavior between solution and film is not fully understood yet, but it suggests that when some *β*_2_ phase is present in solution, it acts as a seed for the film morphology.

**Fig. 5:**
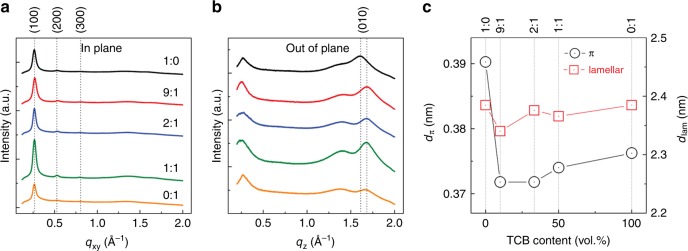
Grazing incidence wide-angle X-ray scattering  (GIWAXS) of thin films. GIWAXS of D-PDPP4T-HD thin films cast from different chloroform:1,2,4- trichlorobenzene (CF:TCB) solutions. **a** In-plane profile. **b** Out-of-plane profile. **c** Lamellar spacing (*d*_lam_) and π-stacking distance (*d*_π_). Note that the scale in panel **c** is different for *d*_π_ and *d*_lam_

Grazing incidence wide-angle X-ray scattering (GIWAXS) is employed to gain structural information of the two aggregated phases including in-plane organization and orientational texture. In order to increase the scattering intensity, drop-casting is utilized to deposit thin films. We emphasize that drop-cast films depict identical polymer aggregation characteristics in the UV-vis-NIR spectra as spin-coated ones (Fig. 4 and Supplementary Fig. 16). All drop-cast films exhibit similar surface topographies with granular features, determined by tapping-mode atomic force microscopy (AFM, Supplementary Fig. 17). The GIWAXS 1D profiles are shown in Fig. 5a, b. In the in-plane profiles, strong lamellar (100), (200), and even (300) peaks are evident for all samples. In the out-of-plane profiles, the (010) peak is seen for all samples. These results demonstrate that D-PDPP4T-HD is self-organized with a predominant face-on packing on the surface, independent of the CF:TCB ratio. Quantitative analysis is performed by assessing the lamellar spacing (*d*_lam_) and π-stacking distance (*d*_π_), as shown in Fig. 5c. All films exhibit almost identical *d*_lam_ values between 2.34 and 2.38 nm. On the contrary, the film processed from pristine CF (1:0) displays slightly larger *d*_π_ (0.39 nm) compared to the other films (~0.37 nm). This can be attributed to the less extended conformation of the main chain in pristine CF, as simulated in Fig. 3. Moreover, in the presence of TCB, *d*_π_ remains almost unchanged with TCB content. We conclude that both *β*_1_ and *β*_2_ phases have face-on packing orientation, and their *d*_π_s are slightly different. Another difference is shown in the orientational distribution. The (100) interlayer reflection intensity profile is plotted as pole figure for the azimuthal angle between −90° and 90°. From this plot it is possible to determine the proportion of the face-on orientation (Supplementary Fig. 18). The trend of *γ*_face_ reveals a slightly higher face-on population in the *β*_1_ phase than in the *β*_2_ phase.

**Fig. 6:**
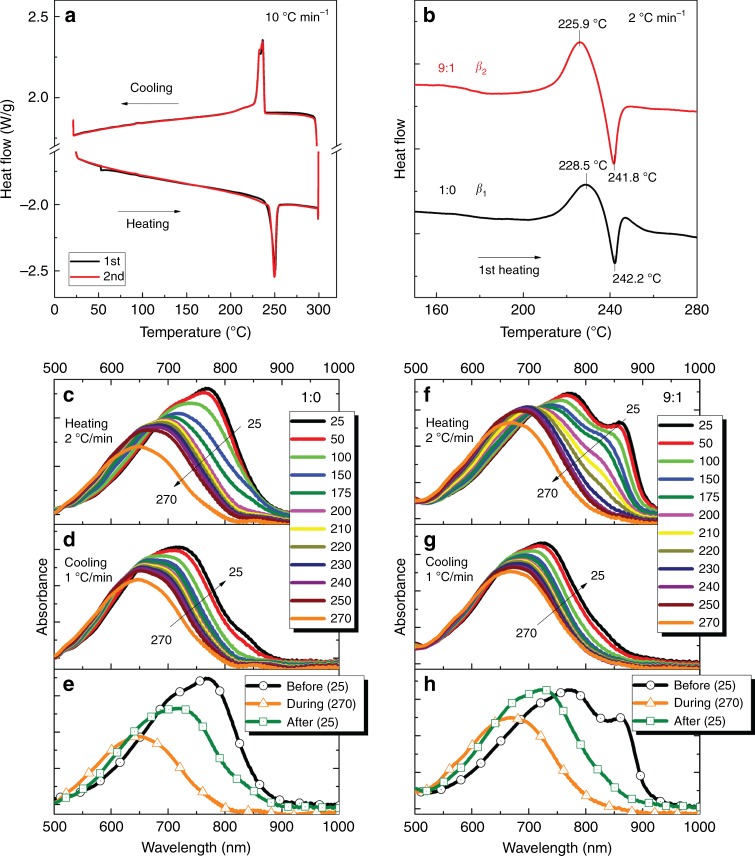
Thermal behavior of polymorphs. **a** Standard DSC thermograms of D-PDPP4T-HD precipitated from chloroform in methanol. The scanning rate is 10 °C min^−1^. **b** The first heating DSC thermogram for D-PDPP4T-HD re-dissolved and dried from different solvents. The scanning rate is 2 °C min^−1^. **c**, **d**, **e**, **f**, **g**, **h**, In situ optical absorption during heating and cooling of D-PDPP4T-HD thin films spin-coated from CF:TCB 1:0 (**c**, **d**, **e**) and 9:1 (**f**, **g**, **h**). N_2_ atmosphere is utilized to protect the polymer films. The heating and cooling rates are 2 °C min^−1^ and 1 °C min^−1^, respectively. The bottom panels compare the optical absorption before and after thermal annealing

Thermal properties of two different aggregated phases of D-PDPP4T-HD are investigated by differential scanning calorimetry (DSC). First, the polymer precipitated from CF in methanol, defined as the standard sample, is measured as control with scan rates of 10 and 40 °C min^−1^ (Fig. 6a and Supplementary Fig. 19). In the heating cycle, a melting temperature (*T*_m_) of 249.6 °C is found; in the cooling process, two close recrystallization peaks at 236.2 and 233.5 °C are observed (Fig. 6a). To measure DSC of the *β*_1_ and *β*_2_ aggregated phases, a simple method is used to process the polymer from the different solvents (Supplementary Fig. 20). Briefly, the inside and bottom walls of a 10-mL glass vial are first coated with poly(acrylic acid) (PAA). After the PAA solidification, a concentrated solution of D-PDPP4T-HD with various solvents is poured into the coated vial, and then dried under vacuum. Consequently, the free-standing D-PDPP4T-HD is obtained by dissolving PAA with Milli-Q water. On the basis of this approach, DSC samples with *β*_1_ or *β*_2_ phases are fabricated for CF:TCB 1:0 or 9:1, and the first heating cycle in the DSC is recorded with slower scanning rate (2 °C min^−1^), as shown in Fig. 6b. In comparison to the standard sample, both *β*_1_ and *β*_2_ phases exhibit a cold crystallization transition (*T*_c_) with peak positions at 228.5 and 225.9 °C. For this *T*_c_ process, the enthalpy of *β*_2_ is 40% higher than that of *β*_1_. Note that both aggregated phases display a similar *T*_m_ of ~242 °C, which is 8 °C lower compared to the precipitated polymer.

Further investigation on thermal properties of the two aggregated phases is conducted through in situ UV-vis-NIR spectroscopy by heating/cooling D-PDPP4T-HD thin films under nitrogen atmosphere. The heating and cooling rates are 2 and 1 °C min^−1^, respectively, and the maximum heating temperature reaches 270 °C. Upon annealing, the 0–0 peak and 0–1 vibronic shoulders of the *β*_1_ phase blue shift and their intensities gradually drop (Fig. 6c). At 250 °C (>*T*_m_), these two signals are replaced by one new main peak. At 270 °C, this new peak is shifted to around 645 nm, resembling the *α* phase in solution (Fig. 1a and Supplementary Fig. 2). The cooling process results in a red shift of absorption spectra and the re-appearance of the 0–0 and 0–1 vibronic features at 720 nm and 660 nm (Fig. 6d). A direct comparison of the absorption spectra before and after annealing is shown in Fig. 6e and reveals that thermal cycling from *β*_1_ → *α* → *β*_1_ is partially reversible during the time scale of the experiment, but ultimately results in a ~50-nm blue shift. Interestingly, a tiny fraction of the *β*_2_ phase is generated at around 850 nm during crystallization. Compared to the *β*_1_ phase, the thermal cycling of the *β*_2_ phase exhibits a substantially lower reversibility. The absorbance at 870 nm vanishes upon heating and is only partly restored in the cooling cycle, as shown in Fig. 6f, g, h and Supplementary Fig. 21. We find that faster heating/cooling rates result in more recovery of the *β*_2_ phase (Supplementary Figs. 21 and 22). If the *β*_1_ and *β*_2_ phases are heated to a fully disordered *α* phase upon annealing to 270 °C, the two samples are expected to form the same state upon returning to 25 °C, because the starting point at 270 °C is the same. This is exactly what is observed, especially for the slower heating and cooling experiment in which the sample is longer above the melt transition where the chains lose their ordered structure. The result that an ordered phase only partly reappears upon cooling from the melt can be attributed to the fact that the formation of a semi-crystalline phase requires polymer chains to realign from the disordered melt. This is hampered by the reduced mobility of polymer chains in the solid films when the temperature drops below the crystallization temperature (233–236 °C, Fig. 6a). The fact that cooling from the melt produces more *β*_1_ phase than *β*_2_ phase is likely a kinetic effect. In solution the *β*_2_ phase forms also more slowly (Supplementary Fig. 13). It is also consistent with the idea that *β*_2_ is the more ordered phase (because it is a lower optical band gap).

### Effect of polymorphism in transistors and solar cells

**Fig. 7:**
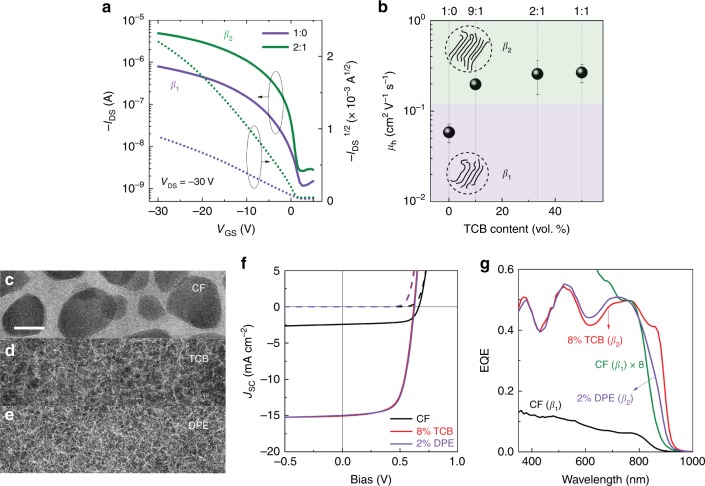
Field-effect transistors and solar cells of the two polymorphs. **a** Transfer characteristics of D-PDPP4T-HD field-effect transistors for the *β*_1_ and *β*_2_ polymorphs processed from chloroform:1,2,4-trichlorobenzene (CF:TCB) solutions (1:0) and (2:1). **b** Average (6 devices) field-effect mobility as a function of TCB content. Error bars are standard deviation. **c**, **d**, **e** TEM images of D-PDPP4T-HD:[70]PCBM blends with various cosolvents. All images share the same scale bar of 200 nm. **f** *J-V* characteristics of D-PDPP4T-HD:[70]PCBM solar cells under simulated AM1.5 G (100 mW cm^−2^) illumination (solid lines). The dash lines indicate the dark current. **g** Corresponding EQE spectra

To investigate the impact of polymorphism of D-PDPP4T-HD on the charge transport in electronic devices field-effect transistors (FETs) were used with a bottom-contact top-gate architecture. The FETs were fabricated on silicon wafers with thermally grown SiO_2_, a spin-coated D-PDPP4T-HD layer from mixed CF:TCB solutions, a CYTOP insulator layer, and a silver gate contact. To remove residual solvent, especially TCB, all polymer films are annealed at 100 °C for 0.5 h in a nitrogen glovebox, which has little effect on film morphology and molecular ordering of conjugated polymers (Fig. 6)^41,42^. Fig. 7a exhibits the transfer characteristics of both aggregated phases at a drain voltage (*V*_DS_) of −30 V. The drain current (*I*_DS_) of the *β*_1_ phase (from CF:TCB 1:0) is lower than that of *β*_2_ phase (from CF:TCB 2:1) and the saturated hole mobility (*μ*_h_) is 0.058 ± 0.014 cm^2^ V^−1^ s^−1^ for *β*_1_ and 0.26 ± 0.11 cm^2^ V^−1^ s^−1^ for *β*_2_, when averaged over six devices. The on/off ratio (*I*_on_/*I*_off_) of about 10^3^ and threshold voltage (*V*_T_) of circa + 3 V are similar for the *β*_1_ and *β*_2_ phases. Fig. 7b shows the effect of the CF:TCB ratio in the solution used to cast the film on the hole mobility. When this ratio exceeds 2:1, the charge transport remains almost unchanged with a mobility on the order of 0.25 cm^2^ V^−1^ s^−1^. Compared to *β*_1_, this 4-fold higher mobility of the *β*_2_ phase is consistent with its reduced π stacking distance as inferred from GIWAXS and the longer conjugation length as inferred from UV-vis-NIR absorption spectroscopy.^43,44^

It is also of interest to evaluate if the two semi-crystalline polymorphs *β*_1_ and *β*_2_ can be identified in donor-acceptor bulk-heterojunction organic photovoltaic (OPV) cells with D-PDPP4T-HD as the donor polymer and [6,6]-phenyl-C_71_-butyric acid methyl ester ([70]PCBM) as acceptor material. To study this, conventional configuration (ITO/PEDOT:PSS/D-PDPP4T-HD:[70]PCBM/LiF/Al) solar cells are fabricated. *β*_1_–only devices fabricated using pure CF afford a very low short-circuit current density (*J*_sc_, integrated from the external quantum efficiency (EQE) with AM1.5 G solar spectrum) of 2.6 mA cm^−2^ and a power conversion efficiency (PCE) of only 1.2%, due to a coarse phase separation between D-PDPP4T-HD and [70]PCBM caused by spinodal liquid-liquid decomposition during drying^45^ (Fig. 7c, f, g and Supplementary Table 5). Using 1,2-dichlorobenzene (*o*-DCB) as cosolvent in CF, it is possible to retain a *β*_1_–only polymorph, but *o*-DCB is not capable of eliminating the spinodal phase separation in the D-PDPP4T-HD:[70]PCBM blend and the PCE remains low (Supplementary Figs. 23 and 24). In solar cells processed with TCB as cosolvent spinodal demixing is fully suppressed and the *β*_2_ phase is present. After optimization, the TCB concentration for the best device is 8% (Supplementary Fig. 25). As shown in Fig. 7f, the current density of solar cells processed from CF with 8% TCB is almost 5-fold higher than that processed from neat CF, resulting in a significant improvement in PCE from 1.2% to 6.4%. Transmission electron microscopy (TEM) images reveal the fibrillar microstructure (Fig. 7d), which facilitates exciton diffusion and charge separation. Furthermore, the EQE in the wavelength range from 820 to 900 nm reaches 0.4, which originates from *β*_2_ phase (Fig. 7g). Diphenyl ether (DPE) as co-solvent (2% in CF) is also capable of suppressing coarse phase separation and generating the *β*_2_ phase, but the EQE at 860 nm (EQE_860_) is only half of the TCB (8% in CF) sample, as shown in Fig. 7g and Table 1. The resultant 2% DPE device provides a PCE of 6.2%, in agreement with our previous report^31^. This confirms that the *β*_2_ phase has a significant contribution to the OPV performance. The somewhat higher *V*_oc_ found for *β*_1_-phase solar cells compared to *β*_2_-phase devices (0.66 vs. 0.62 V), is consistent with the higher ionization potential for *β*_1_ (*IP* = 4.88 eV) than for *β*_2_ (*IP* = 4.80 eV) inferred from ultraviolet photoemission spectroscopy (UPS) (Supplementary Fig. 26).

**Table 1:** Optical, structural, thermal, and electrical properties of the two polymorphs

Property		*β*_1_	*β*_2_
*λ*_0–0_ (nm)	Absorption^a^	770	870
	PL^b^	860	890
*E*_g_^c^ (eV)		1.42	1.37
IP (eV)^d^		4.88	4.80
*d* (nm)	*d*_π_^e^	0.39	0.37
	*d*_lam_^f^	2.4	2.4
Polymer orientation (*γ*_face_^g^, %)		77	74
*T* (°C)	*T*_c_^h^	228.5	225.9
	*T*_m_^i^	242.2	241.8
Enthalpy^j^ (J g^−1^)		22.3	31.4
*μ*_FET_^k^ (cm^2^ V^−1^ s^−1^)		0.058 ± 0.014	0.26 ± 0.11
*V*_oc_^l^ (V)		0.66	0.62
		(0.663 ± 0.000)	(0.621 ± 0.002)
*J*_sc,sr_^m^ (mA cm^−2^)		2.6	15.5
		(2.5 ± 0.1)	(15.1 ± 0.5)
PCE^n^ (%)		1.2	6.4
		(1.1 ± 0.0)	(6.2 ± 0.1)
EQE_860_^o^		0.01	0.41

^a^UV-vis-NIR absorption

^b^Photoluminescence

^c^Optical bandgap

^d^Ionization potential from UPS

^e^π–π stacking distance

^f^Lamellar spacing

^g^Percentage of face-on orientation

^h^Cold crystallization temperature

^i^Melting point

^j^Values are for cold crystallization process and obtained from Fig. 6b

^k^Hole mobility based on the field-effect measurement

^l^Open-circuit voltage, average and standard deviation from five cells between parenthesis

^m^Short-circuit current density determined by integrating the EQE spectrum with AM 1.5 G spectrum

^n^Power conversion efficiency

^o^EQE at 860 nm

Two other well-known cosolvents for DPP polymers, 1,8-diiodooctane (DIO) and 1-chloronaphthalene (CN), are also tested for polymorph control. In both cases, the EQE values in the 820 to 900 nm spectral range are relatively pronounced, suggesting the presence of *β*_2_ phase (Supplementary Fig. 27). More interestingly, by comparing all five different cosolvents, it seems that the fibrillar microstructure and the *β*_2_ phase appear in the photoactive layer simultaneously, resulting in improved device performance (Supplementary Figs. 24 and 27).

### Generality and mechanism of polymorphism

We are in the process of further establishing the structural features of the polymer that enable the *β*_1_ and *β*_2_ polymorphism by varying type and length of the side chains. At present we are looking further into which combination of side chains (linear, branched, branching position, length, number and attachment position of side chains) for the PDPP4T backbone provides a similar polymorphism. Preliminary results identify the *β*_1_–*β*_2_ polymorphism in three other DPP polymers for different linear side chains on the DPP core (Supplementary Fig. 28). However, we have not been able to achieve a similar solvent induced *β*_1_ → *α* → *β*_2_ transition for a PDPP4T polymer with branched 2-ethylhexyl side chains on the DPP unit and on the two adjacent thiophene units, or for a PDPP4T derivative that has 2-decyltetradecyl side chains on the DPP unit and an unsubstituted 4T segment. This demonstrates that the substitution effects are subtle and that establishing structural-property relations requires further investigation. Without doubt solvent quality plays a critical role in chain conformation of conjugated polymers^46,47^, and the appearance of the *β*_2_ phase in this study is associated with the balance between molecule-molecule and molecule-solvent interactions. We have tested if the Hansen solubility parameters, which describe the dispersive (*δ*_D_), polar (*δ*_P_) and hydrogen-bonding (*δ*_H_) forces between polymers and solvent molecules, can provide an interpretation for the solvent-related polymorphism^48,49^. Besides CF and TCB, we also conducted absorption measurements for D-PDPP4T-HD in 1,2-dichlorobenzene, 1-chloronaphthalene and 1,1,2,2-tetrachloroethane (TCE), benzal chloride and (2-chloroethyl)benzene (Supplementary Fig. 29 and Supplementary Table 6). The results show that for several solvents with Hansen parameters that are similar to those of TCB, *β*_2_ formation is not commonly observed in solution, while it is observed in TCE which has parameters closer to CF. Clearly, further efforts are required to understand the mechanism and the origin of polymorphism in conjugated polymers.

## Discussion

Polymorphism of the aggregated phases of a low-bandgap conjugated polymer, namely D-PDPP4T-HD, is identified in solution and in the solid state. The utilization of TCB as solvent or co-solvent results in the formation of a second aggregated phase (*β*_2_) with a distinct absorption peak at around 870 nm. Luminescence lifetimes of 29 ps, 78 ps, and 129 ps are determined by time-resolved PL spectroscopy for *α*, *β*_1_, and *β*_2_, respectively. The PL quantum yield of the *β*_2_ phase is more than 30-fold higher than that of *β*_1_. In the solid state, the aggregated phase, observed from solution, is retained. The spectroscopic, structural, thermal properties and electronic device performance of thin films with two different aggregated phases have been explored, as summarized in Table 1. (1) Compared to *β*_1_, *β*_2_ has a new 0–0 absorption peak at 870 nm, accompanied by an additional emission peak at 890 nm. The corresponding optical bandgap is decreased to 1.37 eV. (2) Both aggregated phases possess similar microstructures and polymer packing. (3) Cold crystallization is observed for both *β*_1_ and *β*_2_, but with larger enthalpy for *β*_2_. (4) *β*_2_ exhibits higher field-effect mobility in OFETs than *β*_1_, which can be attributed to the reduced *d*_π_. More importantly, the additional absorption of *β*_2_ in the near-infrared region enhances the photovoltaic performance of polymer solar cells.

The strategy for polymorphism control reported in this study is compatible with automated and large scale deposition methods such as ink-printing and roll-to-roll techniques. Interestingly, the results indicate that programmable inks with only one polymer can be designed to pattern different polymorphs for electronic devices, creating location-dependent functionalities. On the other hand, D-PDPP4T-HD can be a good candidate for switchable photodetectors and/or phototransistors in the near-infrared region, due to its tunable ability of light absorption. These results offer us a further understanding of the role of polymer polymorphism in structure-property relations in plastic electronics.

## Methods

### Materials

D-PDPP4T-HD was synthesized according to the literature procedure^31^. In this study, two batches of polymers are used, batch I (*M*_n_ = 71.5 kg mol^−1^ and *M*_w_/*M*_n_ = 2.5) and batch II (*M*_n_ = 63.4 kg mol^−1^ and *M*_w_/*M*_n_ = 2.0), as determined by gel permeation chromatography (GPC) at 140 °C on a PL-GPC 120 system using a PL-GEL 10 mm MIXED-C column with 1,2-dichlorobenzene (*o*-DCB) as the eluent and polystyrene as internal standards. Before GPC measurement, polymer was dissolved in *o*-DCB at 140 °C for 1 h. Such minor difference in molecular weight has no effect on the polymorphism of D-PDPP4T-HD, as shown in Supplementary Fig. 30. Batch I is the one used in our previous report^31^. In this study, batch I is used for GIWAXS samples, and batch II for the other experiments and measurements. The similar performance is also apparent from the reproducibility of the photovoltaic performance compared to the previous study.

### Optical spectroscopy

UV-vis-NIR absorption spectroscopy was conducted on a PerkinElmer Lambda 1050 spectrophotometer at various temperatures for both solution and solid states. Steady-state photoluminescence spectra were recorded though an Edinburgh Instruments FLSP920 double-monochromator luminescence spectrometer equipped with a nitrogen-cooled near-IR sensitive photomultiplier (Hamamatsu).

### Time-resolved photoluminescence

For time-resolved photoluminescence measurements the samples were excited with the wavelength-tunable output of an Optical Parametric Oscillator (Radiantis Inspire HF-100), itself pumped by the fundamental at 820 nm of a Ti:sapphire fs oscillator (Spectra Physics MaiTai eHP), yielding 100 fs pulses at a repetition rate of 80 MHz. The PL of the samples was collected with an optical telescope consisting of two plano-convex lenses matched in focal length to a spectrograph (PI Spectra Pro SP2300) for wavelength dispersion and detected with a streak camera (Hamamatsu C10910) system with a temporal resolution of 1.4 ps. The PL data was acquired in photon counting mode using the streak camera software (HPDTA) and exported to Origin Pro 2017 for further analysis.

### Spectral weight analysis

To separate the absorbance spectra and determine the spectral weight of each polymorph phase, MCR-ALS analysis^46,47^ was applied to an augmented matrix comprised of the absorption spectra of each sample (solvent ratio). Singular value decomposition (SVD) was applied to estimate the number of components present in the data matrix. Several constraints were applied to the analysis such as non-negativity of absorbance and concentration. Furthermore, the spectrum of one component was fixed to the absorption spectrum of *α* (in TCB at 80 °C), while the input spectra of the other two components were kept unknown (NaN). Finally, the input concentration of *β*_1_ was fixed to zero for the 0:1 sample only, while the concentrations of all other data points were kept unknown (NaN). This constraint was applied, as otherwise MCR-ALS outputs the concentration of *α* to zero for the 0:1 sample and overestimates the contribution of *β*_1_, while experimentally emission of *α* in the 0:1 sample is clearly seen from TRPL, indicating the presence of *α* in the sample.

### Computational methodology

To illustrate the influence of solvents on the polymer conformations, all-atom molecular dynamics (MD) simulations were performed by Gromacs-4.6.7 with a modified general AMBER force field (GAFF)^48^. The atomic charges were re-optimized from the restrained electrostatic potentials (RESP) at the HF/6–31 G** level of theory. The torsion potential parameters for the dihedral angles between the conjugated units (i.e., DPP and thiophene moieties) were derived from density functional theory (DFT) calculations at the ωB97XD/6-31 G** level. A cutoff of 1.2 nm was used for all non-bonded interactions. All the MD simulations were carried out under the NPT ensemble at the room temperature and atmosphere pressure with three-dimensional periodic boundary conditions. The Berendsen weak coupling and velocity rescaling thermostat were used to control the pressure and temperature with a coupling time of 1 ps and 0.1 ps, respectively.^49,50^ To construct the polymer solution, a single polymer chain with nine repeat units was first put in a box with the lattice length of 35 nm, then the rest space of the box was filled by solvent molecules. After equilibration, the box length was decreased to 23.04 nm and 25.75 nm for the CF and TCB solutions, respectively. A total simulation time of 30 ns was run and the sampling data were collected every 2 ps from the last balanced time of 10 ns.

### Structural characterizations

Grazing incidence wide-angle X-ray scattering (GIWAXS) was performed under vacuum (~1 mbar) at the DELTA Synchrotron using beamline BL09 with a photon energy of 10 keV. The incident angle of X-ray beam is around 0.1°, above the critical angle of the polymer film but below that of the substrate. The scattering intensity was detected on a 2D image plate (MAR-345), and the software WAXStools was utilized for data processing and analysis^51^.

### Thermal properties

Differential scanning calorimetry (DSC) was determined using a TA Instruments Q2000 DSC in Tzero hermetic pans. Sample preparation from different solvents is described in Supplementary Fig. 20. In situ absorption measurements were performed by fixing a Linkam heating stage into the PerkinElmer Lambda 1050 spectrophotometer with a home-made holder. During the heating/cooling procedures, the samples were protected under nitrogen atmosphere.

### Device fabrication and characterization

A bottom-contact top-gate architecture was utilized for the fabrication of OFET devices. The source and drain electrodes with 60 nm in thickness were deposited by Au evaporation, and the ratio of channel length to width is 1/20. CYTOP insulator was spin-coated on top of D-PDPP4T-HD thin film as dielectric layer, followed by annealing at 100 °C for 1 h. Finally, 50-nm Ag was evaporated as gate electrode. A Keithley 4200-SCS was used for all standard OFET measurement under vacuum.

Pre-patterned indium tin oxide (ITO) substrates (15 Ω per square, Naranjo Substrates) were used for photovoltaic devices with a conventional configuration (ITO/PEDOT:PSS/D-PDPP4T-HD:[70]PCBM/LiF/Al). All photoactive layers were deposited by spin-coating from a chloroform solution with different co-solvents. The active area of the cells was 0.09 or 0.16 cm^2^ as determined by the overlap of the ITO bottom contact and aluminum top contact, and no dependence of photovoltaic performance on the active area was found. The white light (~100 mW cm^−2^) from a tungsten-halogen lamp filtered by a Schott GG385 UV filter and a Hoya LB120 daylight filter was used for illumination. *J−V* characteristics were measured in a N_2_-filled glove box (<1 ppm O_2_, < 1 ppm H_2_O) at ambient temperature, without any preconditioning, using a Keithley 2400 source meter from +2 to −2 V in 0.01 V step size at a rate of 30 ms per data point. Short-circuit currents under AM1.5 G conditions were estimated from the EQE and integration with the AM1.5 G (100 mW cm^−2^) solar spectrum. The EQE was recorded by a lock-in amplifier (Stanford Research Systems SR 830) under simulated 1 sun operation conditions with bias light from a 530 nm high-power LEDs (Thorlabs). As probe light, the light from a 50 W tungsten halogen lamp (Osram64610) was modulated with a mechanical chopper before passing the monochromator (Oriel, Cornerstone 130) for wavelength selection using a 2 mm diameter aperture to illuminate the active area. A calibrated Si cell was used as reference. Polymer solar cells were kept behind a quartz window in a nitrogen filled container.

## Supplementary information

Supplementary Information

Solar Cells Reporting Summary

## Data Availability

All relevant data in this study are available from the corresponding author upon request.
