# Supplementary material for: Impact of polymorphism on the optoelectronic properties of a low-bandgap semiconducting polymer
Source: Nat Commun. 2019 Jun 28;10:2867. doi: 10.1038/s41467-019-10519-z (PMC6599012; doi:10.1038/s41467-019-10519-z)
Supplement: Supplementary file 1 — Supplementary Information [file 41467_2019_10519_MOESM1_ESM.pdf]

**Supplementary Information**

# Impact of polymorphism on the optoelectronic properties of a low-bandgap semiconducting polymer

**Li et al.**

## Supplementary Figures

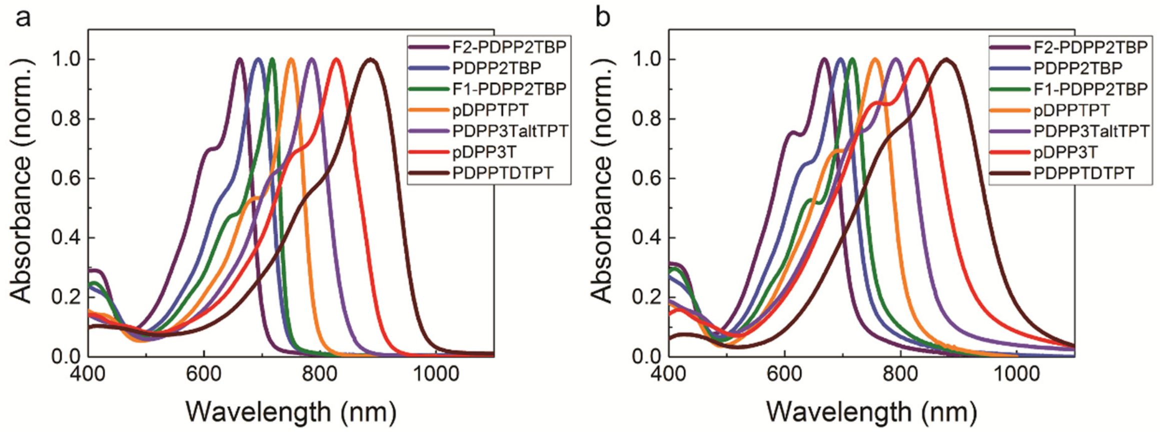


**Supplementary Figure 1 | Optical absorption spectra of diketopyrrolopyrrole polymers.** For a series of diketopyrrolopyrrole (DPP) based polymers the UV-vis-NIR spectra of aggregated phase show a vibronic progression with an intense 0-0 transition at high wavelength and a less intense 0-1 transition at lower wavelength[1-3]. **a**, Spectra recorded for dilute solutions. **b**, Spectra recorded for thin films.

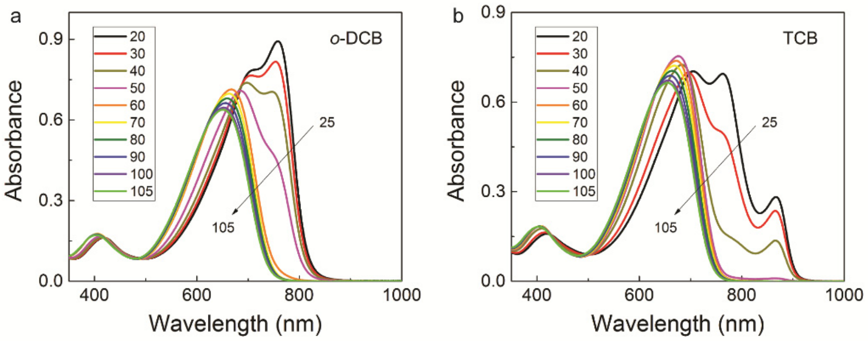


**Supplementary Figure 2 | Temperature dependent UV-vis-NIR absorption in solution. a,** D-PDPP4T-HD in 1,2-dichlorobenzene (*o*-DCB) showing the $\beta_1 \rightarrow \alpha$ transition with increasing temperature. **b**, D-PDPP4T-HD in 1,2,4-trichlorobenzene (TCB) showing the $\beta_2 \rightarrow \alpha$ transition with increasing temperature. The transitions occur in narrow temperature ranges, typical of phase transitions and are followed by smaller gradual spectral shifts to lower wavelengths that can be interpreted as being the result of increased conformational (interring rotation) freedom.

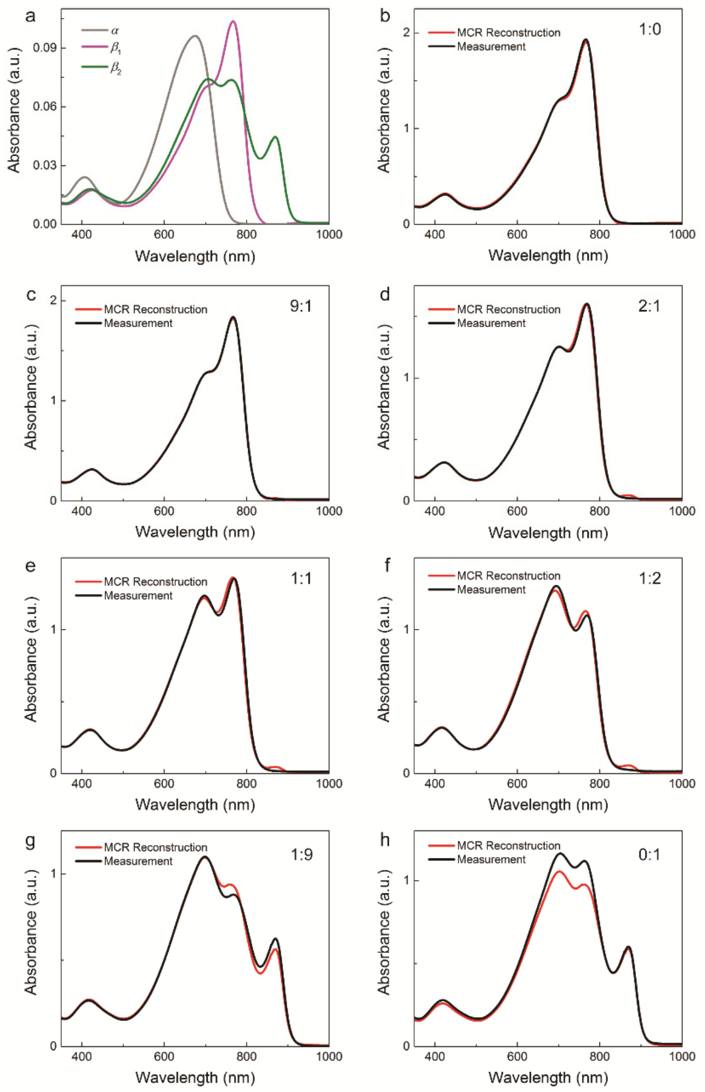


**Supplementary Figure 3 | Deconvolution of $\alpha$, $\beta_1$ and $\beta_2$ components in mixed solutions.** Multivariate curve resolution alternating least squares deconvolution of D-PDPP4T-HD polymorphs in chloroform:1,2,4-trichlorobenzene (CF:TCB) solutions. The absorption spectrum of $\alpha$ (in TCB at 80 °C) is used as input, and the concentration of $\beta_1$ is forced to zero for 0:1. **a**, Deconvoluted spectra for $\alpha$, $\beta_1$ and $\beta_2$ phases. Experimental and reconstructed spectra for D-PDPP4T-HD in CF:TCB at volume ratios: **b**, 1:0; **c**, 9:1; **d**, 2:1; **e**, 1:1; **f**, 1:2. **g**, 1:9; **h,** 0:1

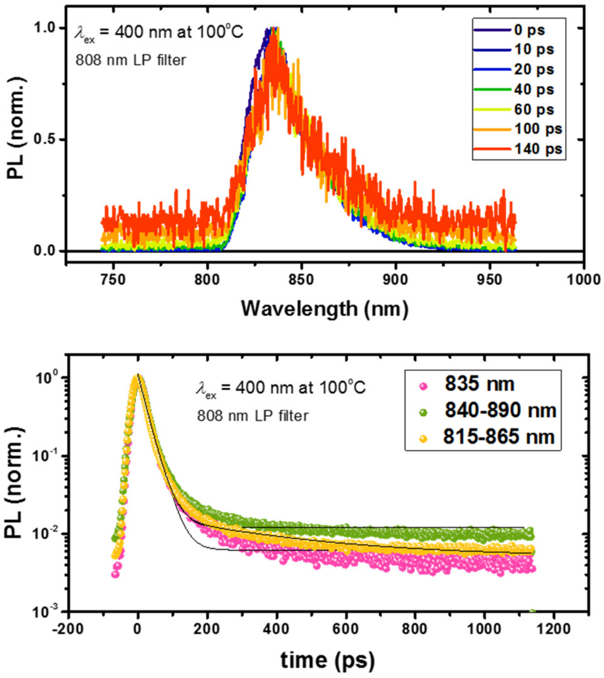


**Supplementary Figure 4 | Time-resolved photoluminescence.** D-PDPP4T-HD in 1,2,4-trichlorobenzene (TCB) solution at 100 °C. Excitation was performed at 400 nm with 80 MHz laser pulse repetition rate. A 808 nm long-pass filter was used to suppress the scattered laser pulse light.

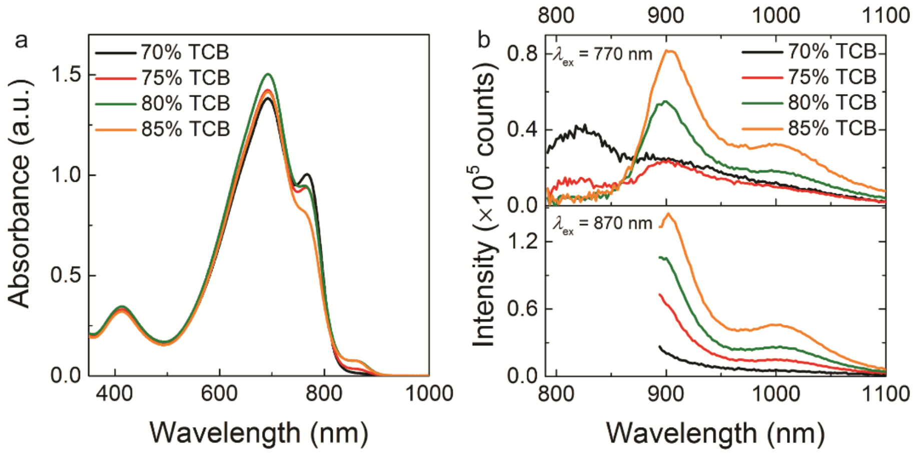


**Supplementary Figure 5 | Optical properties near the critical solvent ratio. a**, UV-vis-NIR absorption spectra of D-PDPP4T-HD in chloroform (CF) with 1,2,4-trichlorobenzene (TCB). **b**, Corresponding steady-state photoluminescence spectra. Starting from 70% TCB, the generation of $\beta_2$ is observed.

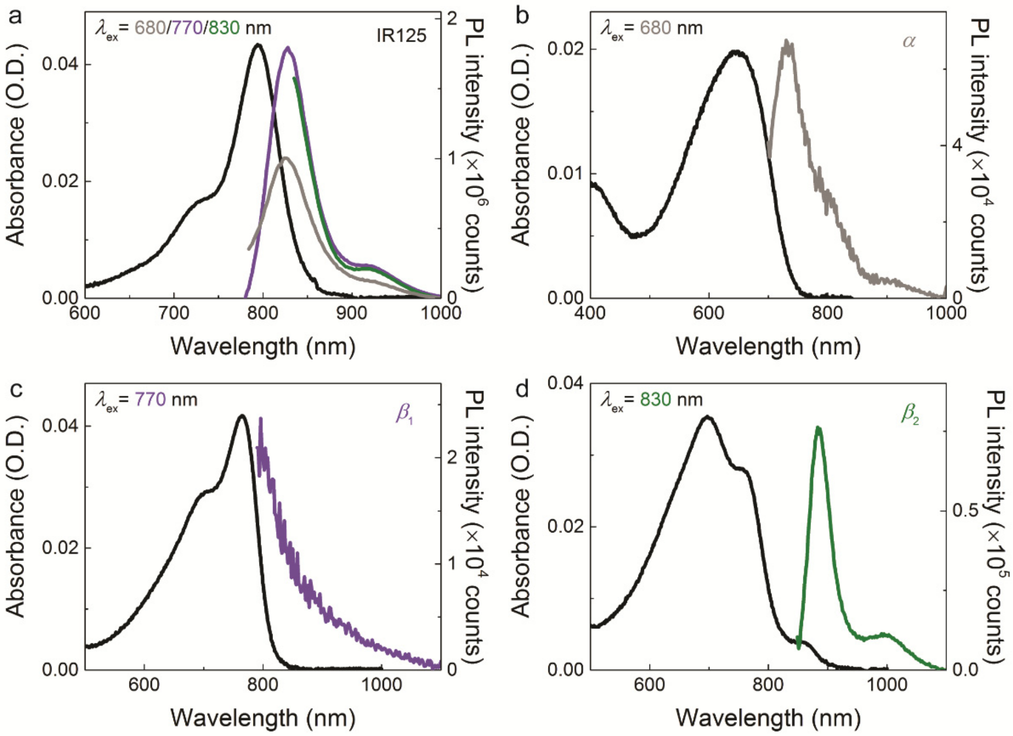


**Supplementary Figure 6 | Determination of luminescence quantum yield of $\alpha$, $\beta_1$ and $\beta_2$.** The optical density is kept below 0.05 to avoid inner filter effects. The reference dye (Indocyanine Green, IR 125) is dissolved in dimethyl sulfoxide (DMSO). $\beta_1$ and $\beta_2$ are generated in neat chloroform (CF) and neat 1,2,4-trichlorobenzene (TCB), respectively

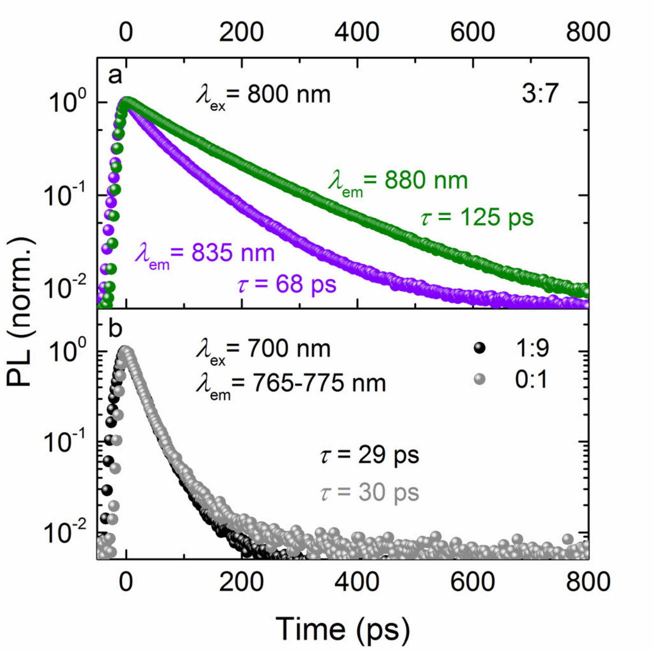


**Supplementary Figure 7 | Time-resolved photoluminescence in solution.** D-PDPP4T-HD in chloroform:1,2,4-trichlorobenzene (CF:TCB) mixtures. **a,** $\beta_1$ region ($\lambda_{em}$=835 nm) and $\beta_2$ region ($\lambda_{em}$=880 nm) of 3:7 solution with $\lambda_{ex}$=800 nm. **b,** $\alpha$ region ($\lambda_{em}$=765-775 nm) of 1:9 (black) and 0:1 (grey) solutions with $\lambda_{ex}$=700 nm and a 750 nm long-pass filter. At the highest TCB concentrations (1:9 and 0:1), the emission kinetics of the $\alpha$ phase ($\lambda_{em}$=765-775 nm) in the presence of $\beta_2$ are detected with 700 nm excitation. The photoluminescence lifetimes of 29 ps for 1:9 and 30 ps for 0:1 are the same as the neat $\alpha$ phase (29 ps, Supplementary Figure 4), indicating that there is also no energy transfer between $\alpha$ and $\beta_2$ under these conditions (Supplementary Figures 7 and 9f,g, Supplementary Tables 2 and 3).

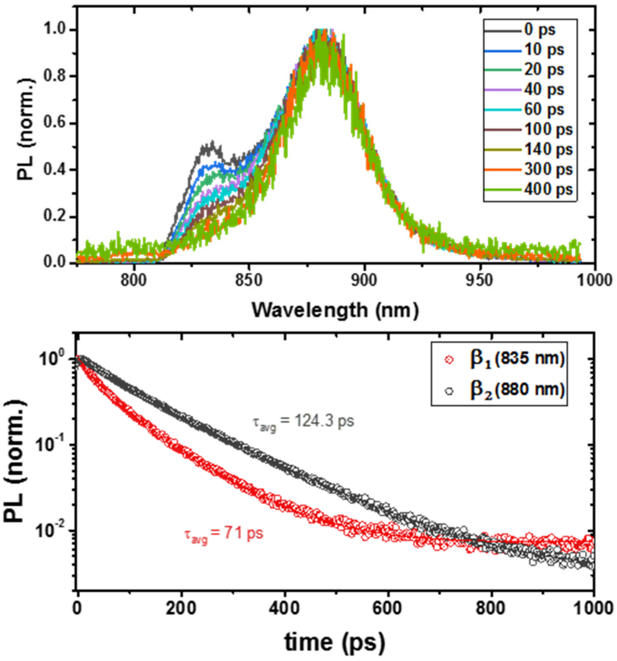


**Supplementary Figure 8 | Time-resolved photoluminescence in solution.** D-PDPP4T-HD is in chloroform:1,2,4-trichlorobenzene (1:3). The bi-exponential fitted decay shows the lifetime of 71 ps for $\beta_1$ ($\lambda_{em}$=835 nm) and 124 ps for $\beta_2$ ($\lambda_{em}$=880 nm), further confirming that there is no energy transfer between $\beta_1$ and $\beta_2$.

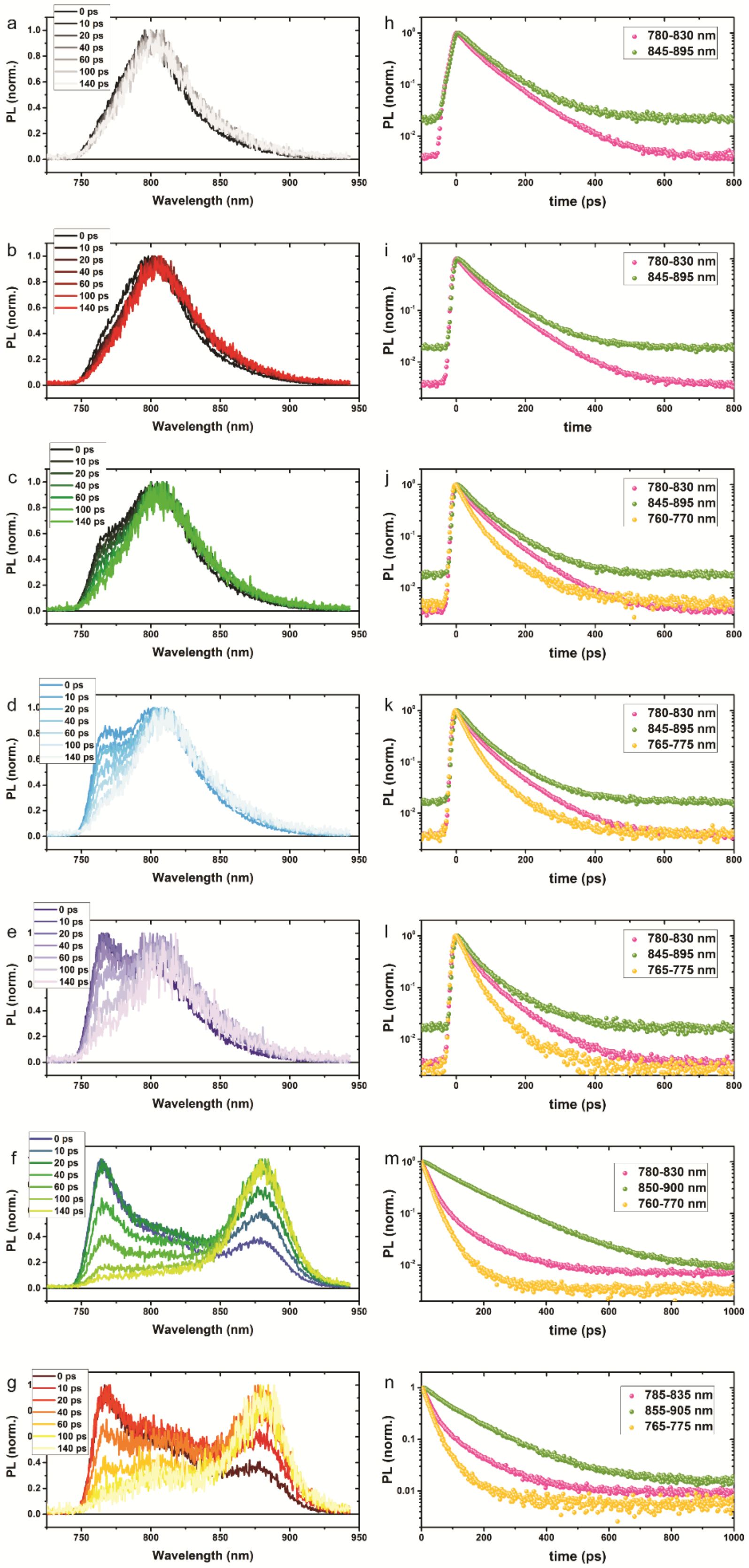
a
b
c
d
e
f
g
h
i
j
k
l
m
n
0 ps
10 ps
20 ps
40 ps
60 ps
100 ps
140 ps
PL (norm.)
Wavelength (nm)
780-830 nm
845-895 nm
760-770 nm
765-775 nm
850-900 nm
785-835 nm
855-905 nm
765-775 nm
time (ps)
time

**Supplementary Figure 9 | Time-resolved photoluminescence of D-PDPP4T-HD.** Normalized PL spectra recorded at various time delays of D-PDPP4T-HD in chloroform:1,2,4-trichlorobenzene (CF:TCB) solvent mixtures at volume ratios of **a**, 1:0; **b**, 9:1; **c**, 2:1; **d**, 1:1; **e**, 1:2. **f**, 1:9; **g,** 0:1. The polymer concentration is 0.4 μM in all cases. **h,i,j,k,l,m,n** are the corresponding kinetics, with $\alpha$ region integrated between 760 and 770 nm, $\beta_1$ aggregation region integrated between 780 and 830 nm, and $\beta_2$ aggregation region integrated between 850 and 900 nm. Solutions were excited with 700 nm pulses at 80 MHz. A 750 nm long-pass filter was used to cut the scattered laser pump.

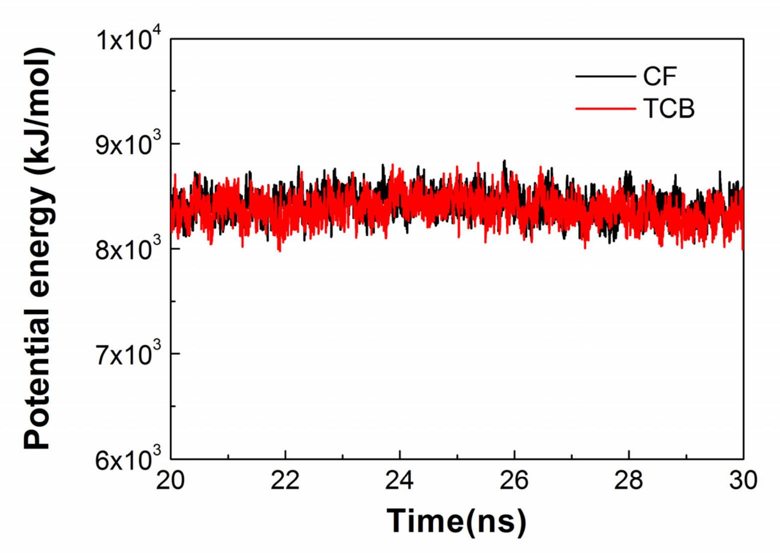


**Supplementary Figure 10 | Potential energy of a single chain in solution.** The potential energy D-PDPP4T-HD nonamer chains in chloroform (CF) and 1,2,4-trichlorobenzene (TCB). In both cases, the potential energy is almost constant, indicating that the system has reached equilibrium in our sampling time.

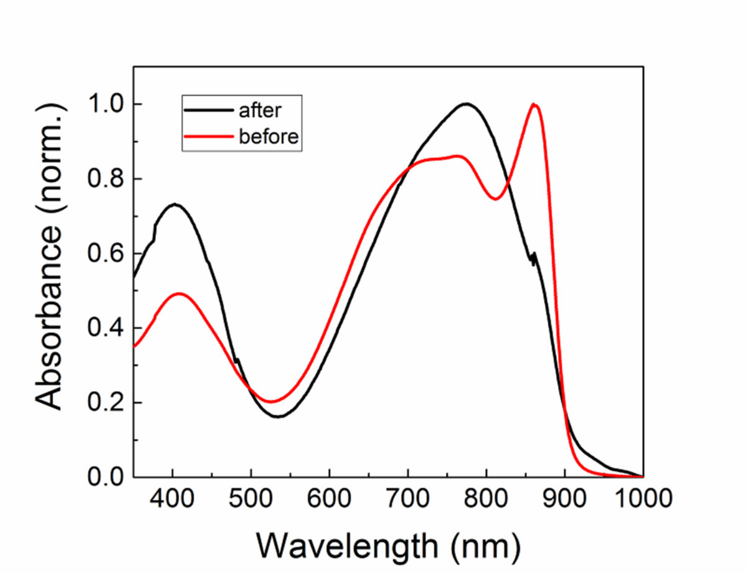


**Supplementary Figure 11 | $\beta_2 \rightarrow \beta_1$ phase transformation in film induced by chloroform.** The absorption spectra of a D-PDPP4T-HD thin film spin-coated from chloroform:1,2,4-trichlorobenzene (CF:TCB) (2:1) before and after phase (polymorph) transformation. The phase transformation is performed by drop-casting CF solvent onto the thin film with substrate temperature of 50 °C.

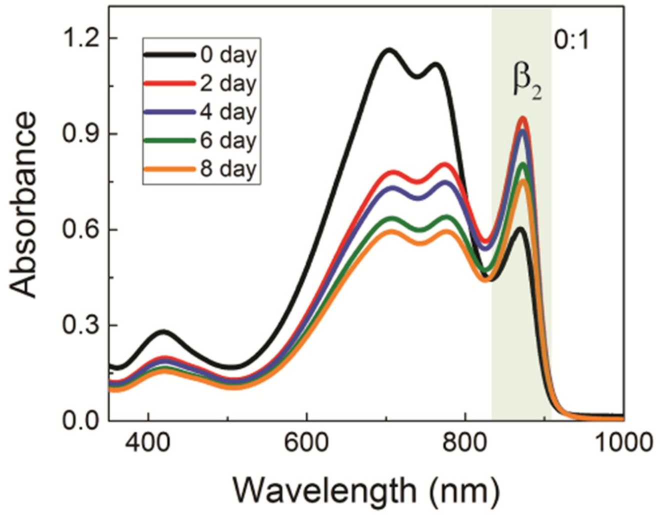


**Supplementary Figure 12 | Aging of D-PDPP4T-HD solutions in 1,2,4-trichlorobenzene.** Change in UV-vis-NIR absorption spectra of D-PDPP4T-HD solutions in pure 1,2,4-trichlorobenzene solution ($\beta_2$) as function of time.

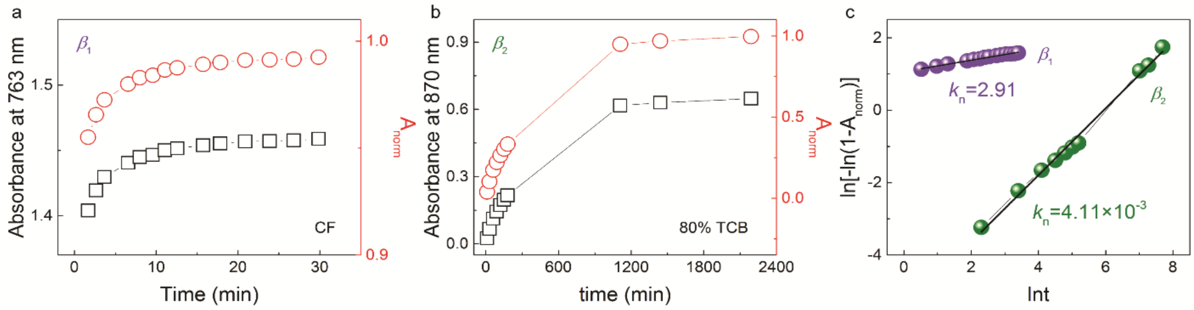


**Supplementary Figure 13 | Kinetics of polymorph crystallization for $\beta_1$ and $\beta_2$. a,b,** Absorbance evolution as a function of time. Two solutions, pure chloroform (CF) and 80% 1,2,4-trichlorobenzene (TCB) in CF, are used to generate $\beta_1$ and $\beta_2$. Both solutions are heated at 90 °C to dissolve polymer, and then placed to room temperature for spontaneous cooling (define time as 0 min at this moment). It is assumed that both solutions reach room temperature after cooling for 2 min, and then UV-vis spectra are recorded. **c,** The absorbance of feature peak (763 nm for $\beta_1$ and 870 nm for $\beta_2$) is proportional to the degree of aggregation, therefore, the fraction of aggregation ($X_t$) is equivalent to the normalized absorbance ($A_{norm} = A/A_{max}$). The Avrami equation[4-5] can be described by $X_t = 1-\exp(-k_n t^n) = A_{norm}$, where $k_n$ represents the Avarmi rate constant involving both nucleation and growth rate parameters, and $n$ denotes the Avrami exponent. By transformation, we get $\ln[-\ln(1-A_{norm})] = \ln k_n + n\ln t$.

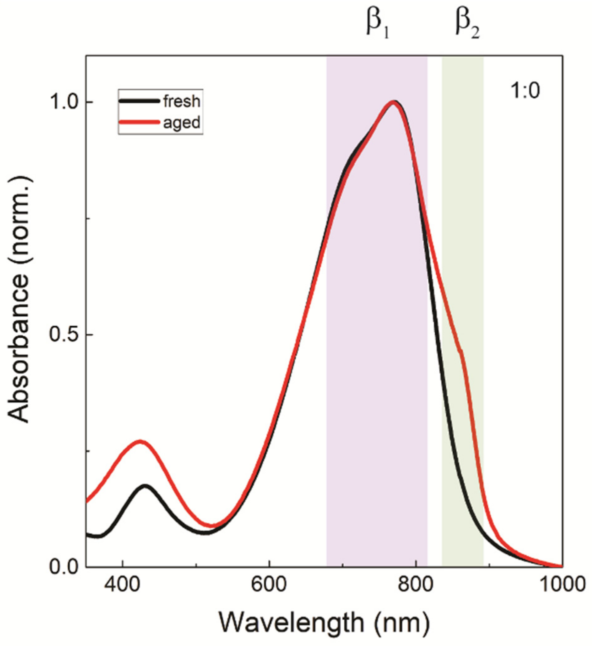


**Supplementary Figure 14 | Influence of aging solution on the aggregation in thin films.** UV-vis-NIR absorption spectra of thin films spin-coated from fresh and aged (2 days) solutions of D-PDPP4T-HD in pure chloroform. The characteristic peaks for the first ($\beta_1$) and the second ($\beta_2$) aggregated phases are indicated by purple and green frames, respectively.

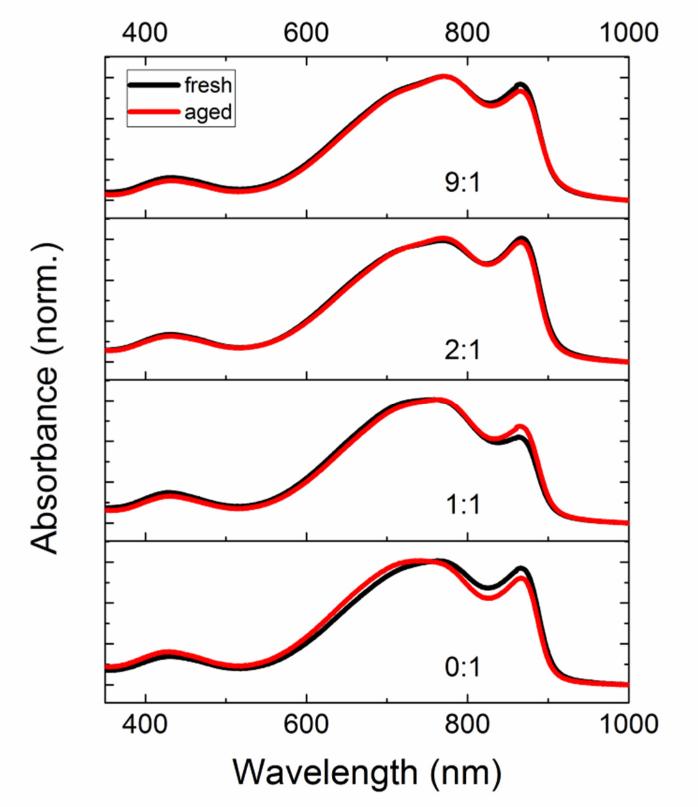


**Supplementary Figure 15 | Influence of aging solutions in CF:TCB on the aggregation.** UV-vis-NIR absorption spectra of thin films spin-coated from fresh and aged (2 days) solutions of D-PDPP4T-HD in chloroform:1,2,4-trichlorobenzene mixtures. The effect of aging on the aggregation in thin films is minimal.

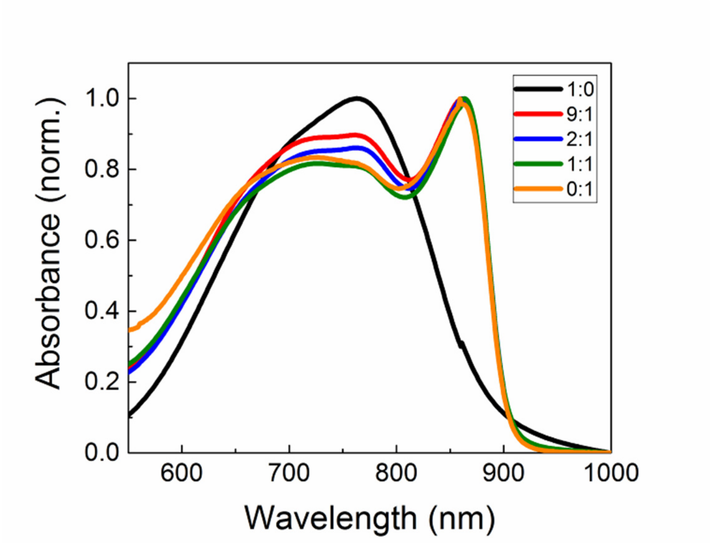


**Supplementary Figure 16 | UV-vis-NIR absorption of D-PDPP4T-HD drop-cast films.** UV-vis-NIR absorption spectra of D-PDPP4T-HD thin films drop-cast from solutions with different chloroform:1,2,4-trichlorobenzene ratios.

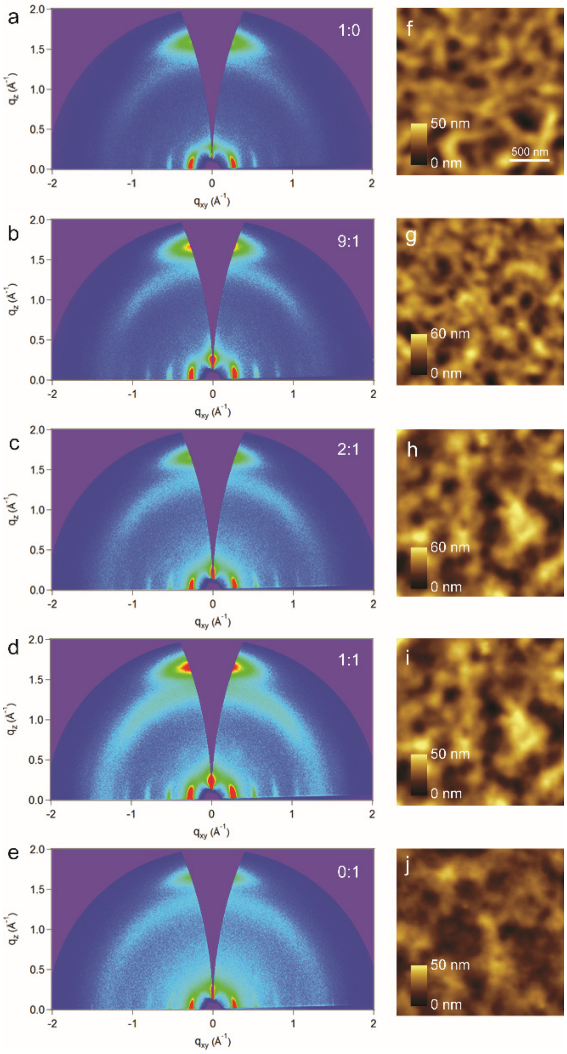


**Supplementary Figure 17 | Microstructure characterization of D-PDPP4T-HD thin films. a-e**, 2D GIWAXS patterns of D-PDPP4T-HD thin films drop-cast from solutions with different chloroform:1,2,4-trichlorobenzene ratios. **f-j**, The corresponding AFM height images. GIWAXS data are processed by the WAXStools software[6].

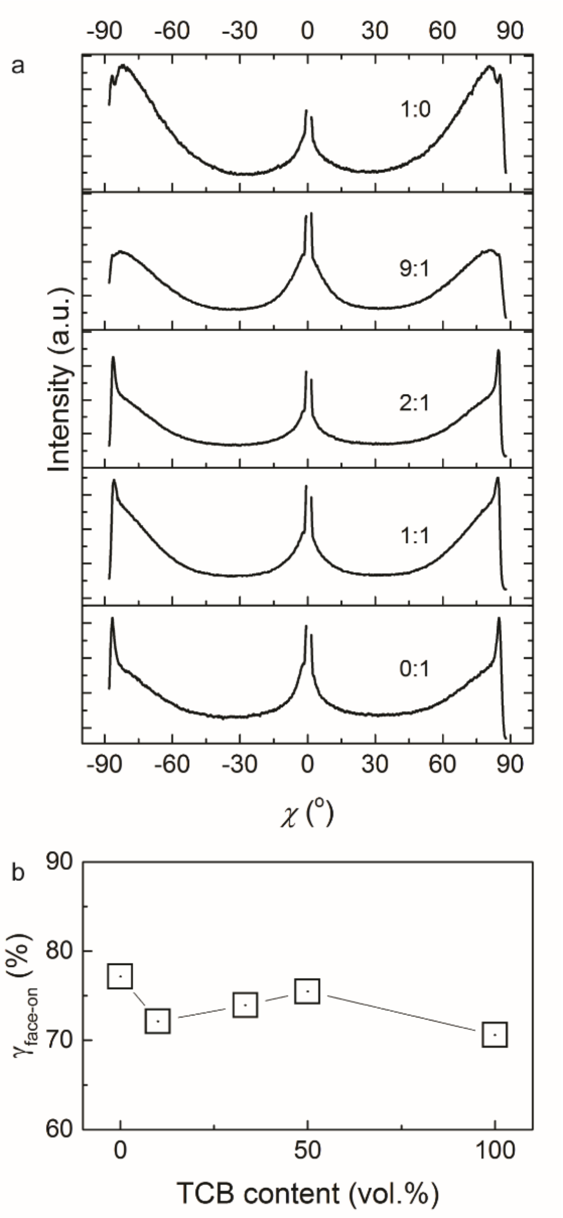


**Supplementary Figure 18 | Azimuthally integrated intensity at (100) reflection. a,** The integrated area between −30° to 30° is defined as edge-on, while the rest integrated area is associated with face-on organization (the range from −2° to 2° is excluded for integration due to the discontinuous profiles). **b**, The ratio of face-on orientation ($\gamma_{face-on}$) as a function of 1,2,4-trichlorobenzene (TCB) content. Care must be taken when using data to estimate the proportion of face-on orientation, since the incident angle and film roughness have significant impact on the reflection intensity. Therefore, such analysis only provides qualitative comparison between samples.

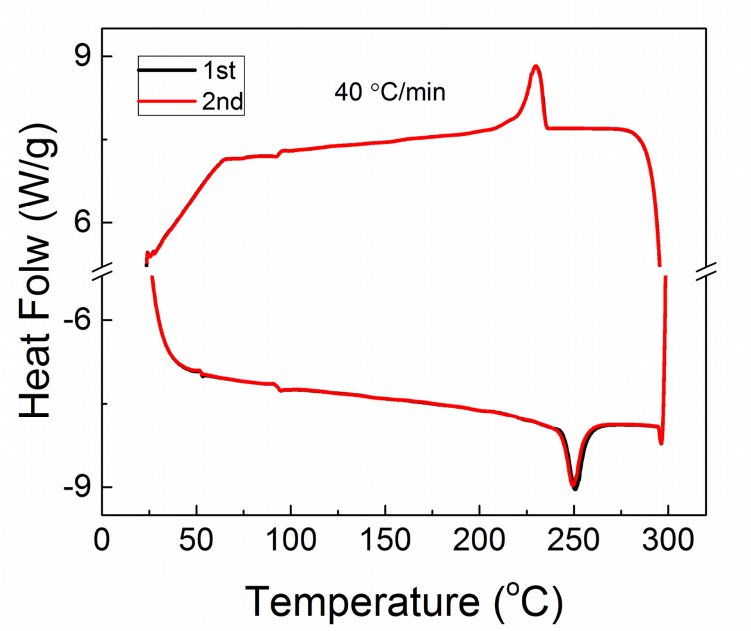


**Supplementary Figure 19 | Differential scanning calorimetry (DSC) of D-PDPP4T-HD.** DSC thermogram measured on D-PDPP4T-HD precipitated in methanol after synthesis. The scanning rate is 40 °C min$^{-1}$.

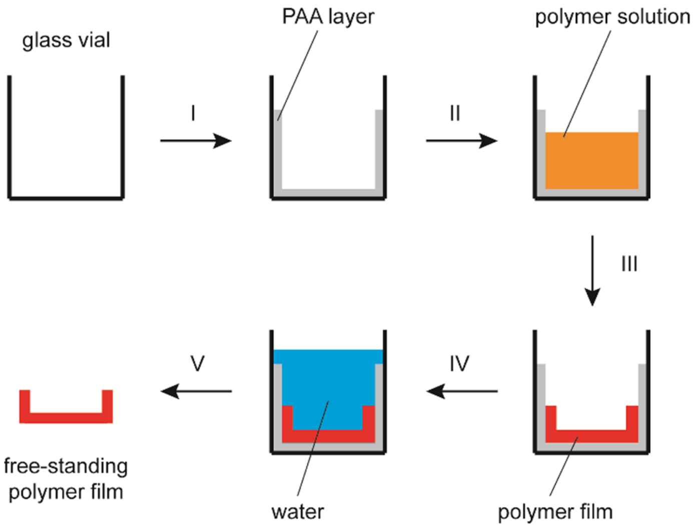


**Supplementary Figure 20 | Sample preparation for differential scanning calorimetry (DSC).** To investigate the thermal properties of two different aggregated phases with DSC, samples were prepared with the following steps. I: Coat poly(acrylic acid) (PAA) layer inside of a glass vial and dry it; II: pour polymer solution (chloroform:1,2,4-trichlorobenzene (CF:TCB)) into coated vial with liquid level below PAA layer; III: put the vial containing solution into vacuum to accelerate solvent evaporation, and then obtain polymer film; IV: pour Milli-Q water into the vial with the liquid level above PAA layer to dissolve PAA; V: wash free-standing polymer film with Milli-Q water for three times and dry it. The choice of CF:TCB 9:1 for $\beta_2$ is due to the high boiling point of TCB, and it took more than 2 h to dry this solution under vacuum. Higher TCB content requires longer drying time.

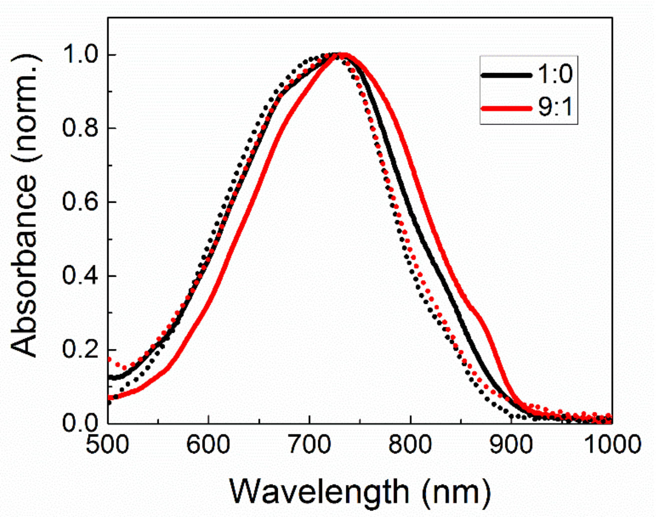


**Supplementary Figure 21 | Thermal history of $\beta_1$ and $\beta_2$ phases.** Comparison of the optical absorption spectra of the $\beta_1$ and $\beta_2$ phases obtained from 1:0 and 9:1 chloroform:1,2,4-trichlorobenzene solutions after annealing at 270 °C and subsequent cooling to 25 °C. The dot line spectra correspond to Figure 6e and 6h in the main text. Solid lines indicate with faster heating (cooling) rate of 5 °C min$^{-1}$ (2 °C min$^{-1}$), corresponding to Supplementary Figure 22.

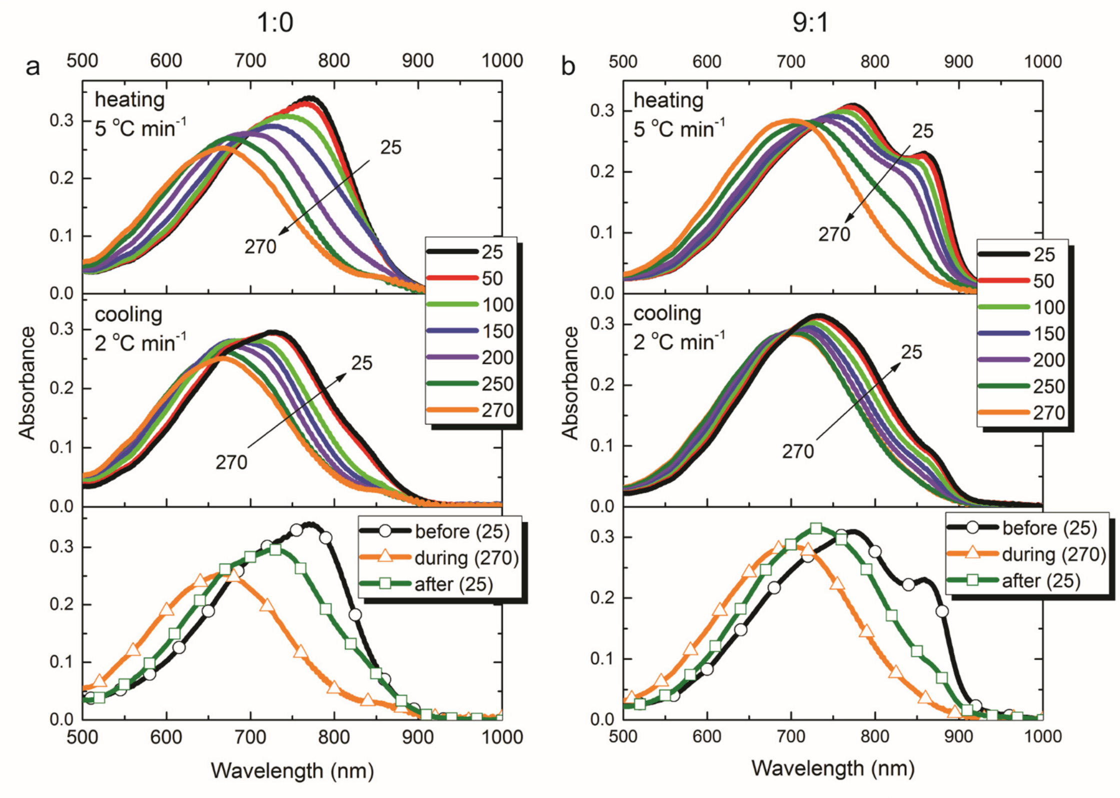


**Supplementary Figure 22 | Thermal behavior of D-PDPP4T-HD polymorphs.** In situ absorption measurements during heating and cooling of D-PDPP4T-HD thin films spin-coated from chloroform:1,2,4-trichlorobenzene 1:0 (**a**) and 9:1 (**b**). $N_2$ atmosphere is utilized to protect polymer films. The heating and cooling rate are 5 °C min$^{-1}$ and 2 °C min$^{-1}$, respectively (i.e. faster than the corresponding Figure 6, shown in the main text).

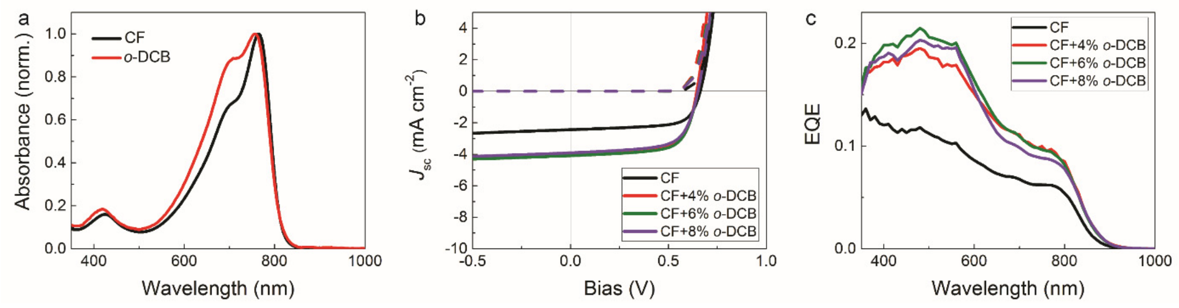


**Supplementary Figure 23 | Optical absorption spectra and solar cell characterization. a,** UV-vis-NIR absorption of D-PDPP4T-HD solution in chloroform (CF) and 1,2-dichlorobenzene (*o*-DCB). No $\beta_2$ phase is generated in *o*-DCB. **b,** *J*-*V* characteristics of D-PDPP4T-HD:[70]PCBM without and with o-DCB as cosolvent. **c,** Corresponding external quantum efficiency (EQE) spectra.

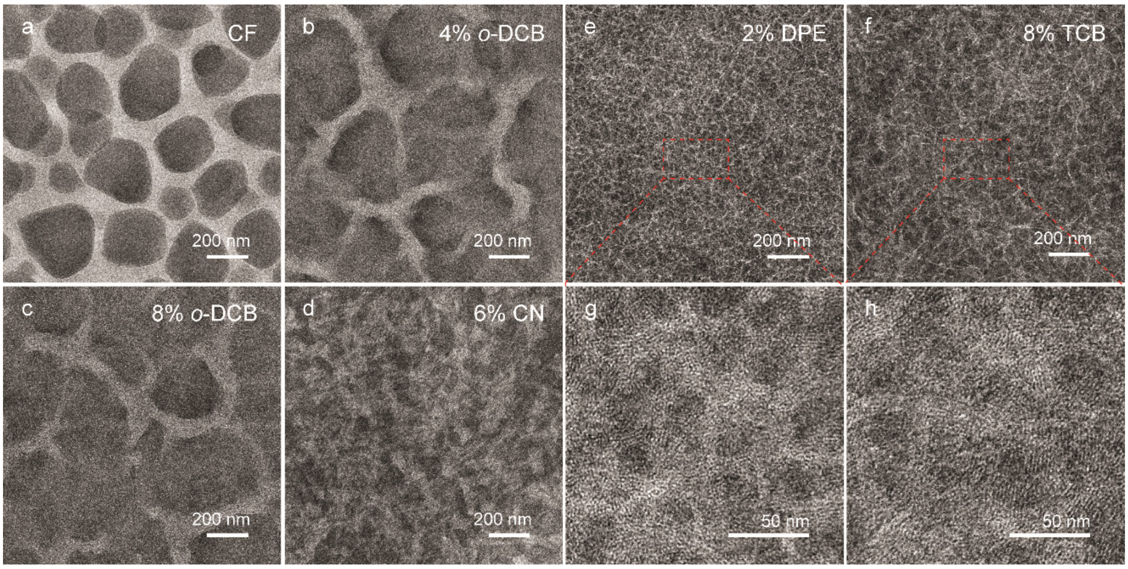


**Supplementary Figure 24 | Influence of cosolvent on the morphology of photoactive layer. a-c,** pure chloroform and 1,2-dichlorobenzene (*o*-DCB) as cosolvent lead to coarse phase separation because of spinodal liquid-liquid decomposition. **d**, 1-chloronaphthalene (CN) as cosolvent results in fibrillar structure with fibril width of 52.3±24.2 nm. **e,f,** Semi-crystalline fibers are observed with fibril width of 11.6±4.0 nm for 2% diphenyl ether (DPE) and 17.9±6.3 nm for and 8% 1,2,4-trichlorobenzene (TCB). **g,h**, fringe structures appear with the identical spacing of around 2.1 nm, in agreement with lamellar spacing obtained from grazing-incidence wide-angle X-ray scattering in Figure 5.

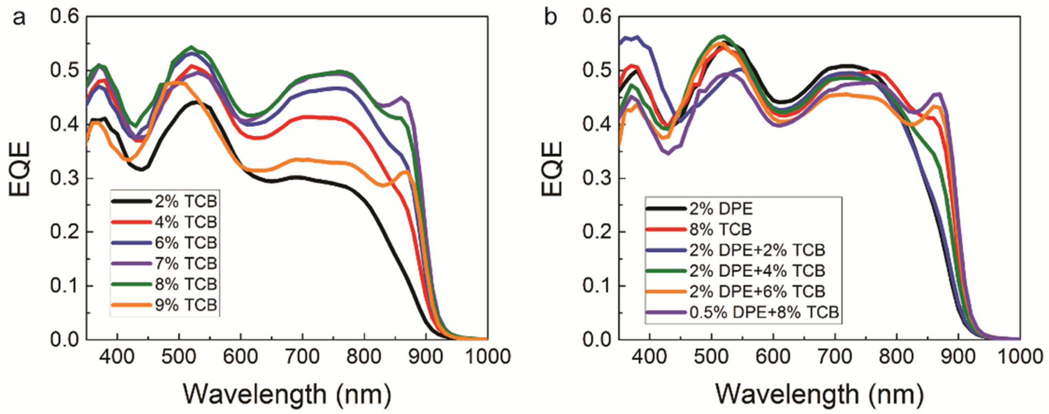


**Supplementary Figure 25 | External quantum efficiency (EQE) spectra of solar cells. a**, For D-PDPP4T-HD:[70]PCBM solar cells fabricated with different amount of 1,2,4-trichlorobenzene (TCB) as co-solvent in chloroform (CF). **b**, D-PDPP4T-HD:[70]PCBM solar cells made with different mixtures of diphenyl ether (DPE) and TCB as co-solvents in CF.

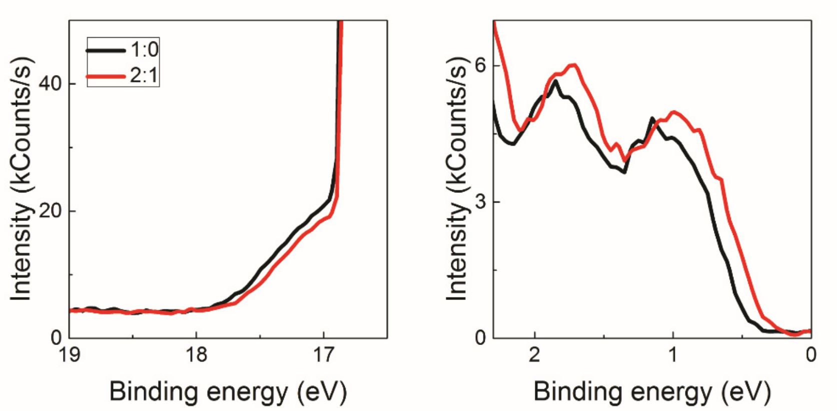


**Supplementary Figure 26 | Ultraviolet photoemission spectroscopy (UPS) of $\beta_1$ and $\beta_2$.** Thin films are fabricated from chloroform:1,2,4-trichlorobenzene solutions of 1:0 and 2:1 for $\beta_1$ and $\beta_2$ phases, respectively. The ionization potentials of 4.88 eV and 4.80 eV are obtained for $\beta_1$ and $\beta_2$ phases, respectively.

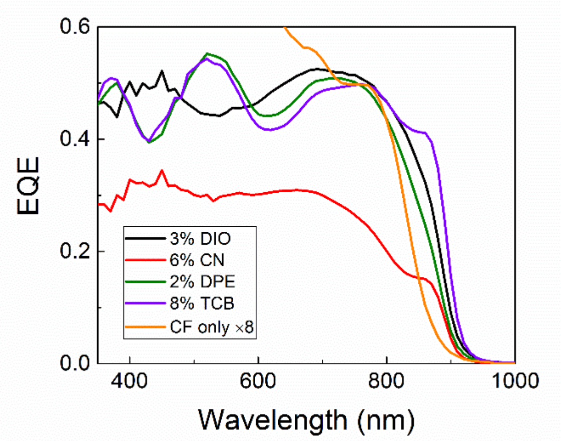


**Supplementary Figure 27 | Influence of cosolvents on external quantum efficiency (EQE).** EQE spectra of D-PDPP4T-HD:[70]PCBM solar cells processed from chloroform (CF) with different cosolvents: diphenyl ether (DPE), 1,2,4-trichlorobenzene (TCB), 1-chloronaphthalene (CN) and 1,8-diiodooctane (DIO). For all cosolvents the contribution of $\beta_2$ phase to EQE is observed. Compared to other cosolvents, lower EQE values of CN are attributed to thicker fibers, as shown in Supplementary Figure 24.

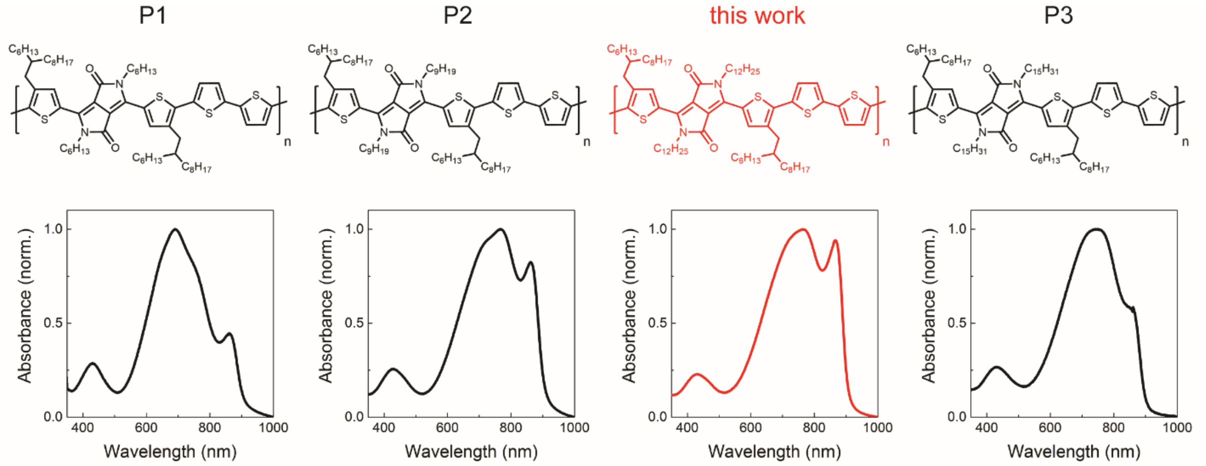


**Supplementary Figure 28 | General behavior of polymorphism for DPP polymers.** The length of the linear side chains on the DPP unit is modified in P1, P2 and P3. Corresponding film absorption spectra confirm the generation of $\beta_2$ phase in all cases with the addition of 1,2,4-trichlorobenzene to the chloroform solution.

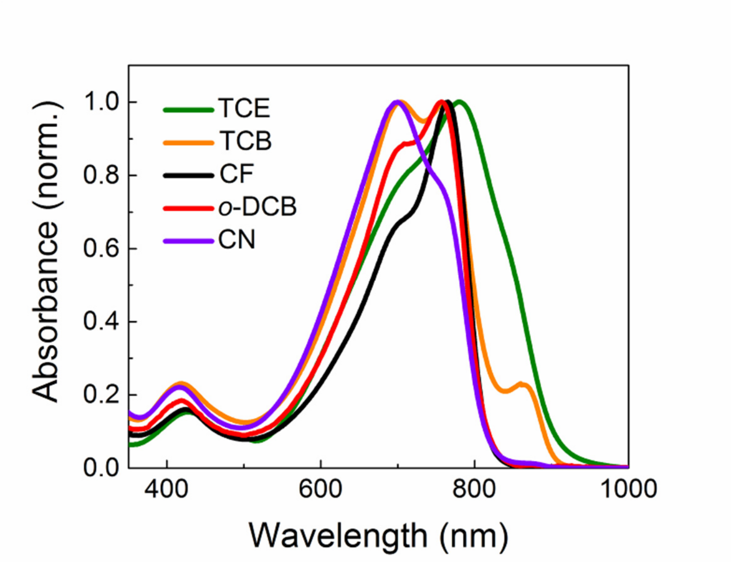


**Supplementary Figure 29 | Absorption spectra of D-PDPP4T-HD in different solvents.** The UV-vis-NIR absorption spectra of dilute solutions of D-PDPP4T-HD in different solvents. Formation of $\beta_2$ phase is apparent in 1,2,4-trichlorobenzene (TCB) and 1,1,2,2-tetrachloroethane (TCE), but not in chloroform (CF), 1,2-dichlorobenzene (*o*-DCB) and 1-chloronaphthalene (CN).

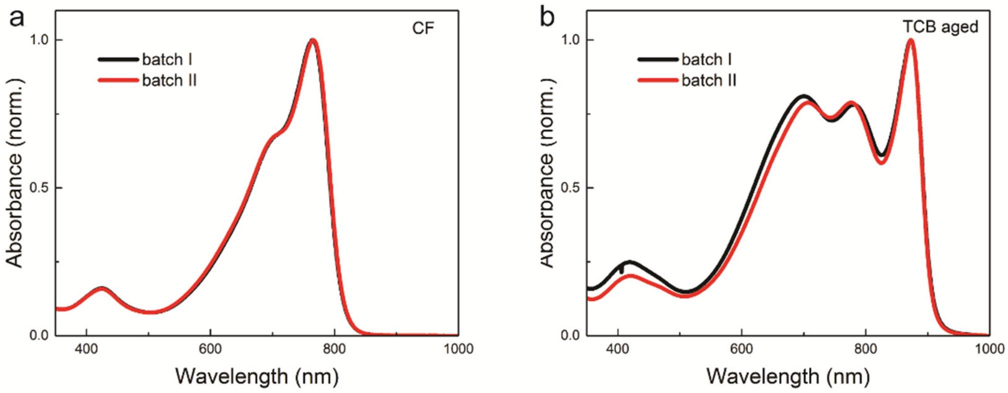


**Supplementary Figure 30 | Effect of molecular weight.** The UV-vis-NIR absorption spectra of two different D-PDPP4T-HD batches (I and II). **a**, In chloroform. **b**, Aged in 1,2,4-trichlorobenzene.

## Supplementary Tables

**Supplementary Table 1 | Excited state lifetimes of polymorphs at the critical solvent ratio.** For D-PDPP4T-HD in chloroform:1,2,4-trichlorobenzene (CF:TCB) solvent mixtures with excitation at 800 nm.

| | $\beta_1$ region (835 nm) | | | | | $\beta_2$ region (880 nm) | | | | |
|---|---|---|---|---|---|---|---|---|---|---|
| | $A_1$ | $\tau_1$ (ps) | $A_2$ | $\tau_2$ (ps) | $\tau_{ave}$ (ps) | $A_1$ | $\tau_1$ (ps) | $A_2$ | $\tau_2$ (ps) | $\tau_{ave}$ (ps) |
| 70%TCB | 0.513 | 37.2 | 0.519 | 98.6 | 68.1 | 0.295 | 61.6 | 0.717 | 150.4 | 124.5 |
| 75%TCB | 0.479 | 32.8 | 0.544 | 104.7 | 71.0 | 0.302 | 65.2 | 0.689 | 150.2 | 124.3 |

Note: $\tau_{ave} = (A_1 \times \tau_1 + A_2 \times \tau_2)/(A_1 + A_2)$

**Supplementary Table 2 | Excited state lifetime of $\alpha$ phase in different wavelength ranges.** For D-PDPP4T-HD in 1,2,4-trichlorobenzene at 100 °C with excitation at 400 nm.

| | $A_1$ | $\tau_1$ (ps) | $A_2$ | $\tau_2$ (ps) | $\tau_{ave}$ (ps) |
|---|---|---|---|---|---|
| 815-865 nm | 1.084 | 24.8 | 0.013 | 358.5 | 28.7 |
| 835 nm | 1.131 | 26.1 | 0 | 0 | 26.1 |
| 840-890 nm | 1.113 | 26.4 | 0 | 0 | 26.4 |

Note: $\tau_{ave} = (A_1 \times \tau_1 + A_2 \times \tau_2)/(A_1 + A_2)$

**Supplementary Table 3 | Excited state lifetime of $\alpha$ in the presence of $\beta_2$.** For D-PDPP4T-HD in different chloform:1,2,4-trichlorobenzene (CF:TCB) solvent mixtures at room temperature with excitation at 700 nm.

| CF:TCB | $A_1$ | $\tau_1$ (ps) | $A_2$ | $\tau_2$ (ps) | $\tau_{ave}$ (ps) |
|---|---|---|---|---|---|
| 1:9 | 1.072 | 29.1 | 0 | 0 | 29.1 |
| 0:1 | 1.040 | 26.4 | 0.027 | 148.2 | 29.5 |

Note: $\tau_{ave} = (A_1 \times \tau_1 + A_2 \times \tau_2)/(A_1 + A_2)$

**Supplementary Table 4 | Summary of kinetics of polymorph crystallization.**

| polymorph | $k_n$ | $n$ | $t_{1/2}$ (min)[a] |
|---|---|---|---|
| $\beta_1$ | 2.91 | 0.17 | $1.1 \times 10^{-4}$ |
| $\beta_2$ | $4.11 \times 10^{-3}$ | 0.92 | 256.5 |

[a]Half-time of crystallization, defined as $(\ln 2/k_n)^{1/n}$.

**Supplementary Table 5 | Contribution of $\beta_2$ phase to the solar cell performance.**

| Cosolvent | $V_{oc}$ (V) | FF | $J_{sc,sr}{}^{m}$ (mA cm$^{-2}$) | PCE (%) | $EQE_{860}$ |
|---|---|---|---|---|---|
| CF[a] only | 0.66 | 0.68 | 2.6 | 1.2 | 0.01 |
| 4% *o*-DCB [b] | 0.65 | 0.70 | 4.2 | 1.9 | 0.02 |
| 6% *o*-DCB | 0.65 | 0.68 | 4.4 | 2.0 | 0.02 |
| 8% *o*-DCB | 0.65 | 0.66 | 4.2 | 1.8 | 0.02 |
| 2% TCB[c] | 0.63 | 0.72 | 10.3 | 4.6 | 0.14 |
| 4% TCB | 0.62 | 0.67 | 13.3 | 5.5 | 0.27 |
| 6% TCB | 0.62 | 0.68 | 14.5 | 6.1 | 0.34 |
| 7% TCB | 0.62 | 0.65 | 15.2 | 6.1 | 0.45 |
| 8% TCB | 0.62 | 0.66 | 15.5 | 6.4 | 0.41 |
| 9% TCB | 0.62 | 0.55 | 11.8 | 4.0 | 0.31 |
| 2% DPE[d] | 0.63 | 0.67 | 14.9 | 6.2 | 0.25 |
| 2% DPE+2% TCB | 0.62 | 0.65 | 14.6 | 5.8 | 0.26 |
| 2% DPE+4% TCB | 0.62 | 0.64 | 15.1 | 6.1 | 0.35 |
| 2% DPE+6% TCB | 0.61 | 0.63 | 15.0 | 5.8 | 0.43 |
| 0.5% DPE+8% TCB | 0.62 | 0.66 | 14.9 | 6.1 | 0.45 |
| 3% DIO[e] | 0.60 | 0.65 | 15.5 | 6.0 | 0.32 |
| 6% CN[f] | 0.65 | 0.62 | 9.0 | 3.6 | 0.15 |

[a]chloroform; [b]1,2-dichlorobenzene; [c]1,2,4-trichlorobenzene; [d]diphenyl ether; [e]1,8-diiodooctane; [f]1-chloronaphthalene.

**Supplementary Table 6 | Hansen solubility parameters for different solvents.[7]**

| Solvent | $\delta_D$ | $\delta_P$ | $\delta_H$ | $V_m$ (cm$^3$/mol)[a] | Observation |
|---|---|---|---|---|---|
| CF[b] | 17.8 | 3.1 | 5.7 | 80.7 | Forms $\beta_1$ phase |
| *o*-DCB[c] | 19.2 | 6.3 | 3.3 | 112.8 | Forms $\beta_1$ phase |
| CN[d] | 19.9 | 4.9 | 2.5 | 136.2 | Forms $\beta_1$ phase |
| TCB[e] | 20.2 | 6.0 | 3.2 | 125.5 | Forms $\beta_2$ phase |
| TCE[d] | 18.8 | 5.1 | 5.3 | 105.2 | Forms $\beta_2$ phase |
| benzal chloride | 19.9 | 6.6 | 2.4 | 134.2 | Reactive, polymer degrades |
| (2-chloroethyl)benzene | 19.3 | 6.3 | 2.2 | 131.5 | Polymers is insoluble |

[a]Molar volume; [b]chloroform; [c]1,2-dichlorobenzene; [d]1-chloronaphthalene; [e]1,2,4-trichlorobenzene; [f]1,1,2,2-tetrachloroethane.

## Supplementary Note 1

The luminescence quantum yield ($\phi$) is determined by comparison of the wavelength integrated intensity of the unknown sample to that of the reference[8]:

$$\phi = \phi_R \cdot \frac{I}{I_R} \cdot \frac{OD_R}{OD} \cdot \frac{n^2}{n_R^2} \quad (1)$$

where $I$ is the integrated emission intensity, OD is the optical density at the excitation wavelength, and $n$ is the refractive index of the solvent used. The subscript R refers to the reference material. Herein, IR 125 (Indocyanine Green) is chosen as the reference with $\phi_R = 23\%$[9]. To avoid inner filter effects, the optical density is kept below 0.05 in all solutions, as shown in Supplementary Figure 7. The refractive index is 1.45 for CF, 1.57 for TCB and 1.48 for DMSO ($n_R$). The resulting $\phi$ is 0.9% for $\alpha$, 0.2% for $\beta_1$ and 6.5% for $\beta_2$.

## Supplementary References


1. Li, W. et al. Wide band gap diketopyrrolopyrrole-based conjugated polymers incorporating biphenyl units applied in polymer solar cells. *Chem. Commun.* **50**, 679–681 (2014).

2. Hendriks, K. H., Heintges, G. H. L., Gevaerts, V. S., Wienk, M. M., Janssen, R. A. J. High-Molecular-Weight Regular Alternating Diketopyrrolopyrrole-based Terpolymers for Efficient Organic Solar Cells. *Angew. Chem. Int. Ed.* **52**, 8341–8344 (2013).

3. Hendriks, K. H., Li, W., Wienk, M. M., Janssen, R. A. J. Small-Bandgap Semiconducting Polymers with High Near-Infrared Photoresponse. *J. Am. Chem. Soc.* **136**, 12130–12136 (2014).

4. Avrami, M. Kinetics of Phase Change. I General Theory. *J. Chem. Phys.* **7**, 1103–1112 (1939).

5. Avrami, M. Kinetics of Phase Change. II Transformation ‐ Time Relations for Random Distribution of Nuclei. *J. Chem. Phys.* **8**, 212–224 (1940).

6. Oosterhout, S. D. *et al*. Mixing Behavior in Small Molecule:Fullerene Organic Photovoltaics. *Chem. Mater.* **29**, 3062–3069 (2017).

7. Hansen, C. M. Hansen solubility parameters: a user's handbook. 2nd ed.; CRC Press: Boca Raton, FL (2007).

8. Lakowicz, J. R. *Principles of Fluorescence Spectroscopy*. 3rd ed.; Springer: Boston, MA (2006).

9. Würth, C., Grabolle, M., Pauli, J., Spieles, M., Resch-Genger, U. Relative and absolute determination of fluorescence quantum yields of transparent samples. *Nat. Protoc.* **8**, 1535–1550 (2013).
