# Supplementary material for: Impact of polymorphism on the optoelectronic properties of a low-bandgap semiconducting polymer
Source: Nat Commun. 2019 Jun 28;10:2867. doi: 10.1038/s41467-019-10519-z (PMC6599012; doi:10.1038/s41467-019-10519-z)
Supplement: Supplementary file 2 — Solar Cells Reporting Summary [file 41467_2019_10519_MOESM2_ESM.pdf]

nature research

Corresponding author(s): Rene Janssen

Solar Cells Reporting Summary

Nature Research wishes to improve the reproducibility of the work that we publish. This form is intended for publication with all accepted papers reporting the characterization of photovoltaic devices and provides structure for consistency and transparency in reporting. Some list items might not apply to an individual manuscript, but all fields must be completed for clarity.

For further information on Nature Research policies, including our data availability policy, see Authors & Referees.

Experimental design

Please check: are the following details reported in the manuscript?

1. Dimensions

Item	Yes/No	Details
Area of the tested solar cells	☒ Yes ☐ No	Methods->Device fabrication and characterization
Method used to determine the device area	☒ Yes ☐ No	Methods->Device fabrication and characterization

2. Current-voltage characterization

Item	Yes/No	Details
Current density-voltage (J-V) plots in both forward and backward direction	☐ Yes ☒ No	Organic solar cells do not have any significant hysteresis
Voltage scan conditions<br>*For instance: scan direction, speed, dwell times*	☒ Yes ☐ No	Methods->Device fabrication and characterization
Test environment<br>*For instance: characterization temperature, in air or in glove box*	☒ Yes ☐ No	Methods->Device fabrication and characterization
Protocol for preconditioning of the device before its characterization	☒ Yes ☐ No	Preconditoning was not used, see Methods->Device fabrication and characterization
Stability of the J-V characteristic<br>*Verified with time evolution of the maximum power point or with the photocurrent at maximum power point; see ref. 7 for details.*	☐ Yes ☒ No	The study is not about stability

3. Hysteresis or any other unusual behaviour

Item	Yes/No	Details
Description of the unusual behaviour observed during the characterization	☐ Yes ☒ No	Organic solar cells do not have any significant hysteresis
Related experimental data	☐ Yes ☒ No	not applicable

4. Efficiency

Item	Yes/No	Details
External quantum efficiency (EQE) or incident photons to current efficiency (IPCE)	☒ Yes ☐ No	Methods->Device fabrication and characterization
A comparison between the integrated response under the standard reference spectrum and the response measure under the simulator	☒ Yes ☐ No	We only discuss the more reliable EQE integrated Jsc in the paper, because Jsc from solar simulators are generally inaccurate without extensive calibration of the light source.
For tandem solar cells, the bias illumination and bias voltage used for each subcell	☐ Yes ☒ No	no tandem cells

5. Calibration

Item	Yes/No	Details
Light source and reference cell or sensor used for the characterization	☒ Yes ☐ No	Methods->Device fabrication and characterization
Confirmation that the reference cell was calibrated and certified	☒ Yes ☐ No	Methods->Device fabrication and characterization

Calculation of spectral mismatch between the reference cell and the devices under test

- [ ] Yes
- [x] No

We do not use Jsc from J-V measurements in this paper

6. Mask/aperture

Size of the mask/aperture used during testing

- [x] Yes
- [ ] No

We use a 2 mm diameter mask (aperture) for the EQE measurements

Variation of the measured short-circuit current density with the mask/aperture area

- [ ] Yes
- [x] No

We did not test this. We used different device areas 0.09 and 0.16 cm2 without seeing any significant difference, see Methods->Device fabrication and characterization

7. Performance certification

Identity of the independent certification laboratory that confirmed the photovoltaic performance

- [ ] Yes
- [x] No

Not relevant for this work, we are not claiming any PCE record

A copy of any certificate(s)
Provide in Supplementary Information

- [ ] Yes
- [x] No

See above

8. Statistics

Number of solar cells tested

- [x] Yes
- [ ] No

5 cells for each, see Table 1

Statistical analysis of the device performance

- [x] Yes
- [ ] No

Yes, see Table 1

9. Long-term stability analysis

Type of analysis, bias conditions and environmental conditions
For instance: illumination type, temperature, atmosphere humidity, encapsulation method, preconditioning temperature

- [ ] Yes
- [x] No

Not the goal of this work.
